# Harvest-Time–Dependent variations in phytochemistry and in vitro bioactivities of *Vitis vinifera* Leaves

**DOI:** 10.1186/s12906-026-05578-x

**Published:** 2026-09-26

**Authors:** Amal M. El-Feky, Noha E. Ibrahim, Wael Mahmoud Aboulthana

**Affiliations:** 1 Pharmacognosy Department, Pharmaceutical and Drug Industries Research Institute, National Research Centre, 33 El Bohouth St. (Former El Tahrir St.), P.O. 12622, Dokki, Giza, Egypt; 2 Microbial Biotechnology Department, Biotechnology Research Institute, National Research Centre, 33 El Bohouth St. (Former El Tahrir St.), P.O. 12622, Dokki, Giza, Egypt; 3 Biochemistry Department, Biotechnology Research Institute, National Research Centre, 33 El Bohouth St., P.O. 12622, Dokki, Giza, Egypt

**Keywords:** Anti-inflammatory, Cytotoxicity, Pigments, Polyphenols, *Vitis vinifera* L

## Abstract

**Background:**

*Vitis vinifera* L. leaves are rich in bioactive phytochemicals, but their composition and biological activities may vary considerably with harvest time. This study investigated harvest-dependent changes in the phytochemical profile and in vitro biological activities of grape leaves.

**Methods:**

Methanolic extracts of *V. vinifera* leaves collected in March, May, and September were evaluated for photosynthetic pigments, total polyphenols, flavonoids, and tannins. HPLC analysis was used to characterize individual phenolic and flavonoid constituents, while major compounds were isolated and structurally identified. The extracts were further assessed for antioxidant and radical-scavenging, antidiabetic, anti-Alzheimer’s, anti-inflammatory, anti-arthritic, and cytotoxic activities.

**Results:**

Harvest time markedly affected the phytochemical composition and biological activities of the leaves. The May-harvested leaves showed the highest levels of total chlorophyll, polyphenols, flavonoids, and tannins, whereas carotenoids were comparatively higher in the September and March samples. HPLC analysis revealed greater accumulation of phenolic and flavonoid constituents in the May extract, with chlorogenic acid, rosmarinic acid, gallic acid, and naringenin among the predominant compounds. Consistently, the May extract exhibited the strongest antioxidant and radical-scavenging activities and showed superior inhibitory effects in the antidiabetic, anti-Alzheimer’s, anti-inflammatory, anti-arthritic, and cytotoxic assays compared with the other harvest periods.

**Conclusion:**

Harvest time substantially influences the accumulation of bioactive constituents and the in vitro biological potential of *V. vinifera* leaves. Among the investigated periods, May appeared to be the most favorable harvest time for obtaining grape leaves enriched in bioactive phytochemicals with promising biological activities, supporting their potential valorization as a value-added viticultural by-product.

**Supplementary Information:**

The online version contains supplementary material available at https://doi.org/10.1186/s12906-026-05578-x.

## Introduction

*Vitis vinifera* L. (Vitaceae) is one of the world's most widely cultivated fruit crops and has substantial economic and agricultural importance. In Egypt, grape cultivation has expanded considerably, making grapevine an important fruit crop for both local production and agricultural value [25, 41, 76]. Although grapes and their products have received extensive scientific attention, grapevine leaves remain comparatively underexploited despite being rich in structurally diverse secondary metabolites.

Grapevine leaves contain phenolic acids, flavonoids, tannins, stilbenes, and other polyphenolic constituents that contribute to their antioxidant and other biological properties [39, 40]. Compounds such as quercetin, kaempferol, rutin, and apigenin derivatives have been reported in grape leaves and are associated with free-radical scavenging and modulation of oxidative and inflammatory processes. Accordingly, grape leaf extracts have demonstrated antioxidant, anti-inflammatory, antihyperglycemic, and neuroprotective activities in experimental models [9, 14, 15] Aboul [2, 69]. These findings support the potential of grape leaves as a source of naturally occurring bioactive compounds.

The accumulation of secondary metabolites in plant tissues is not constant and can be affected by genotype, geographical conditions, temperature, light intensity, water and nutrient availability, agricultural practices, developmental stage, and harvest time [18, 86]. In grapevine leaves, temporal variation in phenolic constituents has also been reported. For example, [37] demonstrated significant changes in kaempferol-3-*O*-glucoside and its aglycone according to harvest period, while [83] reported seasonal variation in the concentrations of bioactive constituents. Such variation may influence not only the phytochemical profile but also the biological performance of the resulting extracts.

Despite these observations, studies integrating harvest-dependent changes in photosynthetic pigments, major phytochemical groups, individual phenolic constituents, and a broad spectrum of in vitro biological activities of *V. vinifera* leaves remain limited. In particular, information identifying a harvest period associated with enhanced accumulation of bioactive constituents and corresponding biological activity is still insufficient. Establishing such relationships could contribute to the rational utilization of grapevine leaves, an abundant agricultural by-product, as a potential source of value-added natural products.

Therefore, the present study investigated the effect of harvest time on the phytochemical composition and in vitro biological activities of *V. vinifera* leaves collected during March, May, and September, representing distinct stages of the growing season. Photosynthetic pigments, total polyphenols, flavonoids, tannins, and individual phenolic and flavonoid constituents were determined, while antioxidant, radical-scavenging, antidiabetic, anti-Alzheimer’s, anti-inflammatory, anti-arthritic, and cytotoxic activities were evaluated. The study aimed to identify harvest-related changes in phytochemical composition and biological potential and to determine the most favorable harvest period for the valorization of grapevine leaves as a source of bioactive compounds.

The novelty of the present study lies in integrating harvest-time-dependent changes in photosynthetic pigments, major phytochemical groups, individual phenolic constituents, and multiple biological activities of *V. vinifera* leaves to identify the harvest period providing the most favorable bioactive profile. Rather than merely confirming the presence of bioactive compounds in grape leaves, this approach provides a practical basis for selecting and standardizing grape-leaf biomass as a potential raw material for future value-added extracts and functional ingredients.

## Materials and methods

### Plant material

Three separate harvest periods (March, May, and September 2024) of red grape (*Vitis vinifera* L.) leaves were collected from a private farm in Wadi El-Natron, El-Behera Governorate, Egypt (30°28′N, 30°30′E). The selected harvest periods corresponded to three major developmental stages of grapevine leaves in the study region: March (early vegetative growth), May (peak vegetative growth), and September (late growth/pre-senescence). These time points were chosen to evaluate seasonal variations in phytochemical accumulation and biological activities throughout the growing cycle. Taxonomic identification was confirmed by Eng. Therese Labib, plant taxonomy consultant at the Ministry of Agriculture. A voucher specimen was deposited in the herbarium of the National Research Center, Cairo, Egypt (Voucher No. M230). Following collection, the leaf samples were thoroughly washed with tap water to remove surface impurities, drained, and air-dried in a well-ventilated shaded environment until complete dryness.

### Chemicals

All chemicals and reagents used in this study were of analytical grade. Methanol, acetone, acetonitrile, and other solvents were purchased from Merck (Darmstadt, Germany). Standards for HPLC analysis and spectrophotometric assays were obtained from Sigma-Aldrich (St. Louis, MO, USA). All solutions were prepared using distilled water.

### Extraction procedure

The dried leaves were separately ground into a fine powder. For each harvest period, 250 g of powdered leaf material was extracted with 100% methanol by cold maceration at room temperature (25 ± 2 °C) for 72 h per extraction cycle. At the end of each 72-h period, the extract was separated from the plant residue by filtration. The residual plant material was then subjected to a second and third extraction under identical conditions (72 h for each cycle). The three filtrates obtained from each extraction replicate were combined and concentrated under reduced pressure using a rotary evaporator (Buchi Rotavapor R-300, Büchi Labortechnik AG, Flawil, Switzerland) [33]. The extraction procedure therefore comprised three successive 72-h maceration cycles, rather than a single 72-h extraction. Three independent extraction replicates (*n* = 3) were performed for each harvest period (March, May, and September). The dry extract yield was calculated as the weight of the dried extract relative to the initial weight of the dried plant material and was expressed as a percentage. The mean extraction yields were 26.84%, 34.15%, and 29.78% for March, May, and September, respectively. The resulting dry extracts were stored at − 20 °C until further phytochemical and biological analyses.

### Determination of photosynthetic pigments

For each harvest period, 0.5 g of dried leaf powder was extracted with 80% acetone under chilled conditions. The homogenate was centrifuged to remove plant debris, and the supernatant was used for spectrophotometric determination of chlorophyll a, chlorophyll b, and total carotenoids. The absorbance was measured using a UV–Visible spectrophotometer (Shimadzu UV-1800, Shimadzu Corporation, Kyoto, Japan) at 664, 651, and 470 nm for chlorophyll a, chlorophyll b, and carotenoids, respectively. Pigment concentrations were calculated according to the equations described by [13]. Total chlorophylls, chlorophyll a/b ratio, total carotenoids, and pigment index (carotenoids/chlorophyll a) were subsequently calculated. All pigment analyses were performed in triplicate (*n* = 3). and the results were expressed as mg pigment/g dry weight (mg/g DW).

### Determination of major phytochemical constituents

For phytochemical quantification, aliquots of the previously prepared and dried crude methanolic extracts obtained according to Sect. "Extraction procedure" were accurately weighed and reconstituted in 80% (v/v) methanol. The use of 80% methanol at this stage was solely for dissolution of the crude methanolic extracts prior to spectrophotometric analysis and did not constitute a separate plant extraction procedure. The solutions were sonicated for 15 min to ensure complete solubilization and centrifuged at 10,000 rpm for 10 min. The resulting supernatants were used for determination of total polyphenolic, flavonoid, and tannin contents.

Total polyphenolic content (TPC) was determined using the Folin–Ciocalteu method [80], while total flavonoid content (TFC) was measured using the aluminum chloride colorimetric method [12]. Total tannins were quantified using the vanillin–HCl assay [16]. All measurements were performed in triplicate (*n* = 3), and results were expressed as mean ± SE.

$$\mathrm{T}\mathrm{P}\mathrm{C}: \mathrm{m}\mathrm{g} \mathrm{g}\mathrm{a}\mathrm{l}\mathrm{l}\mathrm{i}\mathrm{c} \mathrm{a}\mathrm{c}\mathrm{i}\mathrm{d} \mathrm{e}\mathrm{q}\mathrm{u}\mathrm{i}\mathrm{v}\mathrm{a}\mathrm{l}\mathrm{e}\mathrm{n}\mathrm{t}\mathrm{s} \left(\mathrm{G}\mathrm{A}\mathrm{E}\right) \mathrm{p}\mathrm{e}\mathrm{r} 100 \mathrm{g} \mathrm{d}\mathrm{r}\mathrm{y} \mathrm{w}\mathrm{e}\mathrm{i}\mathrm{g}\mathrm{h}\mathrm{t} \left(\mathrm{m}\mathrm{g} \mathrm{G}\mathrm{A}\mathrm{E}/100 \mathrm{g} \mathrm{D}\mathrm{W}\right)$$

$$\mathrm{T}\mathrm{F}\mathrm{C}: \mathrm{m}\mathrm{g} \mathrm{q}\mathrm{u}\mathrm{e}\mathrm{r}\mathrm{c}\mathrm{e}\mathrm{t}\mathrm{i}\mathrm{n} \mathrm{e}\mathrm{q}\mathrm{u}\mathrm{i}\mathrm{v}\mathrm{a}\mathrm{l}\mathrm{e}\mathrm{n}\mathrm{t}\mathrm{s} \left(\mathrm{Q}\mathrm{E}\right) \mathrm{p}\mathrm{e}\mathrm{r} 100 \mathrm{g} \mathrm{d}\mathrm{r}\mathrm{y} \mathrm{w}\mathrm{e}\mathrm{i}\mathrm{g}\mathrm{h}\mathrm{t} \left(\mathrm{m}\mathrm{g} \mathrm{Q}\mathrm{E}/100 \mathrm{g} \mathrm{D}\mathrm{W}\right)$$

$$\mathrm{T}\mathrm{a}\mathrm{n}\mathrm{n}\mathrm{i}\mathrm{n}\mathrm{s}: \upmu \mathrm{g} \mathrm{c}\mathrm{a}\mathrm{t}\mathrm{e}\mathrm{c}\mathrm{h}\mathrm{i}\mathrm{n} \mathrm{e}\mathrm{q}\mathrm{u}\mathrm{i}\mathrm{v}\mathrm{a}\mathrm{l}\mathrm{e}\mathrm{n}\mathrm{t}\mathrm{s} \left(\mathrm{C}\mathrm{E}\right) \mathrm{p}\mathrm{e}\mathrm{r} \mathrm{m}\mathrm{L} \mathrm{e}\mathrm{x}\mathrm{t}\mathrm{r}\mathrm{a}\mathrm{c}\mathrm{t} \left(\mu \mathrm{g} \mathrm{C}\mathrm{E}/\mathrm{m}\mathrm{L}\right)$$

### HPLC analysis of phenolic and flavonoid compounds

High-performance liquid chromatography (HPLC) analysis was performed using an Agilent 1260 Infinity series system equipped with a quaternary pump, autosampler, column thermostat, and diode-array detector (DAD). Separation was achieved using a C18 column (4.6 × 250 mm, 5 μm particle size) [61]. The mobile phase consisted of water (A) and acetonitrile containing 0.05% trifluoroacetic acid (B) at a flow rate of 0.9 mL/min. The gradient elution program was as follows: 0 min (82% A), 0–5 min (80% A), 5–8 min (60% A), 8–12 min (60% A), 12–15 min (82% A), and 15–20 min (82% A). Detection was performed using a diode-array detector at 280 nm. The injection volume was 5 μL, and the column temperature was maintained at 40 °C [24, 34]. Identification of phenolic and flavonoid compounds was based on comparison of retention times and UV spectra with those of authentic standards. The HPLC system was equipped with an Agilent 1260 Infinity UV–Vis detector. All chromatographic analyses were performed in triplicate (n = 3) for each extract to ensure reproducibility.

### Isolation and identification of major compounds

The isolation procedure was undertaken to obtain representative major constituents of *Vitis vinifera* leaf extracts in purified form for phytochemical confirmation and spectroscopic identification. The three extracts obtained from leaves harvested in March, May, and September were initially examined by thin-layer chromatography (TLC) using pre-coated silica gel F254 plates (Merck, 20 × 20 cm, 0.25 mm thickness). Chromatographic development was performed using distilled water:ethyl acetate:formic acid:acetic acid (100:11:11:26, v/v/v), as previously described by [55]. The developed plates were examined under UV light before and after spraying with 1% ethanolic aluminum chloride (AlCl₃) solution. Prominent bands exhibiting characteristic UV responses and/or positive reactions with AlCl₃ were selected for further isolation. The selected bands were carefully collected from the TLC plates and subjected to an additional purification step by TLC using ethyl acetate:methanol:water (100:13.5:10, v/v/v) as the developing system, following [11]. The purified fractions were collected separately and concentrated prior to spectroscopic characterization. The isolated compounds were characterized by mass spectrometry using a Finnigan Model 3200 instrument operated at an ionization energy of 70 eV. Nuclear magnetic resonance (NMR) spectroscopy was performed using JEOL instruments operating at 270 and 400 MHz for ^1^H-NMR and 125 MHz for ^13^C-NMR analyses. The structures of the isolated compounds were assigned based on their spectroscopic data and comparison with previously reported data.

### In vitro biological activities

For in vitro biological assays, dried methanolic extracts were redissolved in methanol to obtain the required working concentrations. The solutions were vortexed, sonicated when necessary, and filtered prior to use. All prepared solutions were freshly made before each experiment and stored under − 20 °C when not immediately used. All phytochemical and biological assays were conducted in triplicate, and the results were expressed as mean ± standard error (SE).

#### Antioxidant and radical scavenging activities

The total antioxidant capacity (TAC) was calculated in accordance with the procedure outlined by [70]. The outcome was represented as mg of gallic acid equivalent per gram of weight. The iron reducing power (IRP) was quantified in µg/mL by employing the method described by [65], with ascorbic acid **(**Sigma-Aldrich, St. Louis, MO, USA**)** acting as the standard.

The evaluation of the radical scavenging activity of 1,1-Diphenyl-2-picryl-hydrazyl (DPPH) was conducted following the procedure described by [72] to compute the inhibition percentage (Inhib. %) of the DPPH radical and the median inhibitory concentration (IC_50_) for each extract examined, using ascorbic acid as a positive control. The 2,2'-azinobis-(3-ethylbenzothiazoline-6-sulfonic acid) (ABTS) experiment was conducted using the method recommended by [10] to determine the IC_50_ of each studied extract and the Inhib. % of the ABTS radical. According to the method demonstrated by [17], the reaction with the Griess Illosvory reagent was used to evaluate the nitric oxide (NO) radical scavenging activity. The Inhib. % of the NO radical and the IC_50_ of each tested extract were calculated. Extract solutions were prepared at concentrations of 25, 50, 100, 200, and 400 μg/mL for determination of radical scavenging activity.

#### Antidiabetic activity

##### Enzymatic assay

The enzyme assay involved the assessment of the inhibition percentage (Inhib. %) for α-amylase from porcine pancreas (EC 3.2.1.1; Sigma-Aldrich, St. Louis, MO, USA) and α-glucosidase enzymes from *Saccharomyces cerevisiae* (EC 3.2.1.20; Sigma-Aldrich, St. Louis, MO, USA), using acarbose as the benchmark drug [24, 34]. The IC_50_ for each extract that was tested was calculated by plotting a curve that correlates a series of sample concentrations with the Inhib. % of the enzyme. Extract concentrations ranging from 31.25–1000 μg/mL were evaluated for enzyme inhibition.

##### Electrophoretic isoenzyme pattern

Vertical slab polyacrylamide gel electrophoresis (PAGE) was applied to investigate the native electrophoretic α-amylase isoenzyme pattern via mini-gel electrophoresis (BioRad, USA), in accordance with the procedure proposed by [73]. The activity of the α-glucosidase enzyme was measured and visualized with Commassie Brilliant Blue (CBB) and compared to acarbose (standard) using the sodium dodecyl sulfate (SDS) electrophoretic α-glucosidase pattern as advised by [52]. The Quantity One program (version 4.6.2) was employed to calculate the relative mobility (Rf), band quantity (Qty), and band percent (B%) of the isoenzyme types identified electrophoretically on the PAGE plate. The percentages of physiological difference (Diff%) and similarity index (SI%) were determined using the formula developed by [62].

#### Anti-Alzheimer activity

The assay involved calculating the Inhib. % of the acetyl cholinesterase (AChE) enzyme (AChE, EC 3.1.1.7) from electric eel, purchased from Sigma-Aldrich (St. Louis, MO, USA) using Ellman's method, with donepezil used as the standard drug [30]. The IC_50_ of each tested extract was calculated. Samples were tested at concentrations between 25 and 800 μg/mL.

#### Anti-arthritic activity

This assay was aimed at determining the Inhib. % of protein denaturation [20] and the activity of the proteinase enzyme [66] with diclofenac sodium serving as a standard non-steroidal anti-inflammatory drug, prepared in line with the methods described by [56]. The IC_50_ for each sample tested was derived by plotting a curve that illustrated a series of sample concentrations against the Inhib. % of the proteinase enzyme.

#### Anti-inflammatory activity

The suppression of two isoenzymes, cyclooxygenase-1 (COX-1) and cyclooxygenase-2 (COX-2) (ovine/human), as well as the 5-LOX enzyme (human recombinant) obtained from Cayman Chemical Company (Ann Arbor, MI, USA), was used to assess the in vitro anti-inflammatory activities. The COX-1 and COX-2 kit was used to assess the Inhib. % of COX-1 and COX-2 [7]. The 5-LOX kit, as suggested by [46], was used to measure the Inhib. % of 5-Lipoxygenase (5-LOX). Using linear regression, the IC_50_ was calculated.

#### Cytotoxic activity

Cytotoxic activities were determined against human hepatocellular (HepG-2), colon carcinoma (CACO-2) and lung cancer (A549) cells using the 3-(4,5-dimethylthiazol-2-yl)−2,5-diphenyl tetrazolium bromide (MTT) assay as suggested by [84]. The cancer cell lines employed in this research were obtained from NAWAH scientific research center. The Inhib. % of cell growth and IC_50_ were calculated using IC_50_ calculation software. According to the published procedures in the literature [44, 67], the enzymatic activities of the various extracts under investigation against caspase-3 and Bcl-2 were assessed within the examined cancer cells (HepG-2, CACO-2, and A549 cells).

### Statistical analysis

All experiments were performed in triplicate, and the results were expressed as mean ± standard error (SE). Statistical analysis was carried out using one-way analysis of variance (ANOVA) followed by Duncan’s Multiple Range Test (DMRT) for post-hoc comparison among the different harvest periods. Differences were considered statistically significant at p < 0.05. Statistical analyses were performed using SPSS software version 26.0 (IBM Corp., Armonk, NY, USA).

**Fig. 1:**
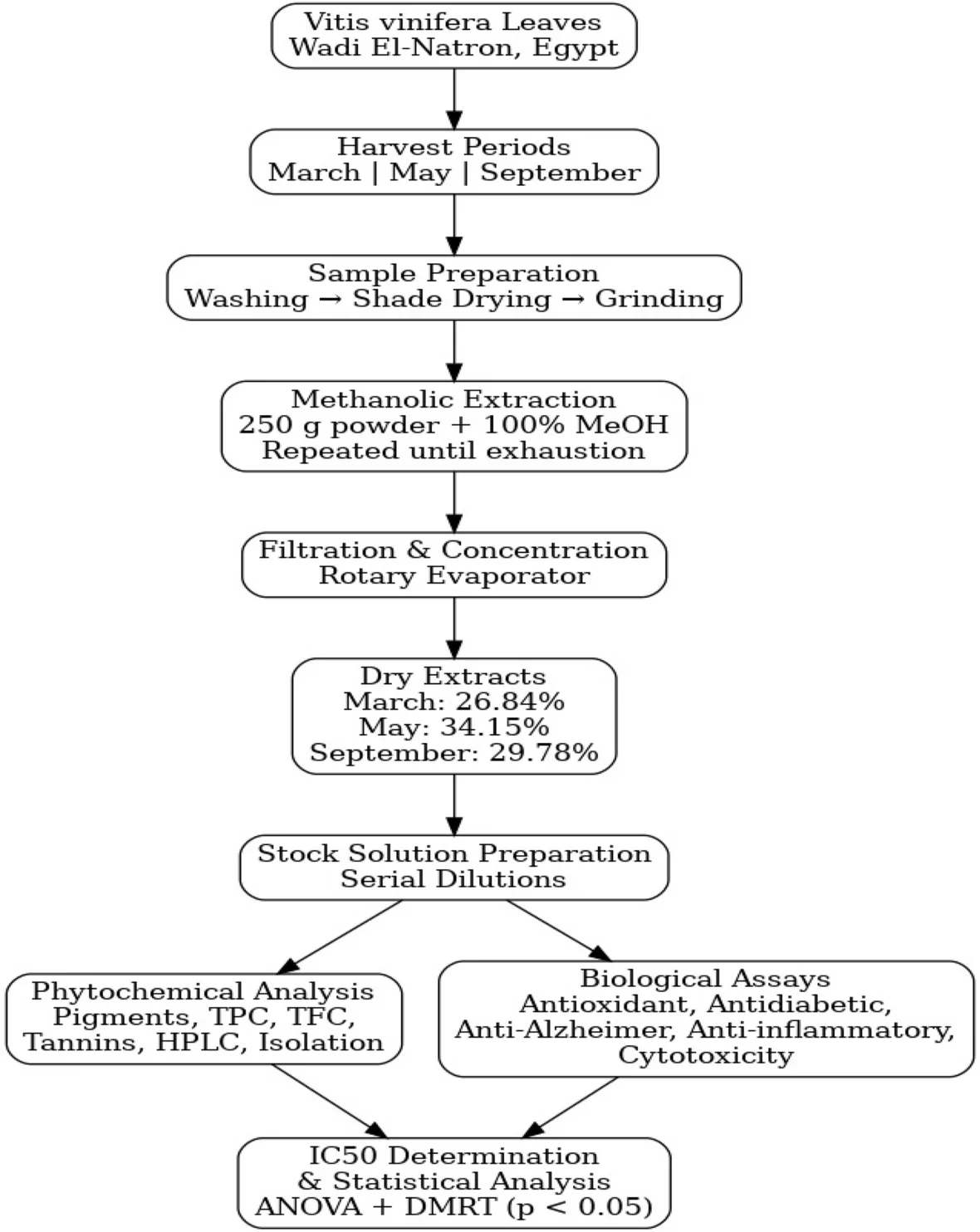
Experimental workflow and sample preparation of *Vitis vinifera* leaves harvested at different growth stages

A schematic workflow illustrating sample collection, extraction, phytochemical characterization, biological activity assessment, and statistical analysis is presented in Fig. 1.

## Results

### Leaf pigments content

Significant seasonal variations were observed in leaf pigment composition **(**Table 1**).** Analysis of the data shows that chlorophyll a and b levels reached their highest values in May, followed by a gradual decline in September, before reaching their lowest concentrations in March. On the other hand, the concentration of carotenoids showed the opposite pattern to that of chlorophyll, reaching its highest point in March (2.95 ± 0.13 mg/g) and its lowest point in May (2.08 ± 0.22 mg/g).

**Table 1:** Pigment contents in extract from *V. vinifera* leaves harvested at three different harvest times

Harvest time	Pigment content (mg/g)					
	**Chlorophyll a**	**Chlorophyll b**	**Chlorophylls a/b ratio**	**Total chlorophylls**	**Total carotenoids**	**Pigment index**
**March**	1.68 ± 0.09^c^	1.32 ± 0.08^b^	1.27 ± 0.10^a^	3.25 ± 0.14^c^	**2.95 ± 0.13**^**a**^	**1.75 ± 0.09**^**a**^
**May**	**2.21 ± 0.13**^**a**^	**1.67 ± 0.10**^**a**^	**1.33 ± 0.08**^**a**^	**3.97 ± 0.09**^**a**^	2.08 ± 0.22^b^	0.94 ± 0.16^c^
**September**	1.97 ± 0.11^b^	1.48 ± 0.21^ab^	1.32 ± 0.11^a^	3.61 ± 0.20^b^	2.14 ± 0.21^b^	1.34 ± 0.10^b^

### The major phytoconstituents

Three harvest periods were used to measure the concentrations of the major phytoconstituents in *V. vinifera* leaves, namely total polyphenols, total tannins, and total flavonoids. As shown in Table 2, the methanolic extract of leaves harvested in May exhibited the highest levels of total polyphenols, tannins, and flavonoids. In contrast, the March-harvested leaves showed the lowest concentrations of these measured phytoconstituents.

**Table 2:** Concentrations of the major active phytoconstituents in extract from *V. vinifera* leaves harvested at three different harvest times

**Harvest time**	**Total Polyphenols** **(**mg gallic acid/100 g**)**	**Total Tannins** **(**μg/mL**)**	**Total Flavonoid** **(**mg quercetin/100 g**)**
March	52.14 ± 0.38^c^	23.17 ± 0.17^c^	13.24 ± 0.10^c^
May	**195.51 ± 1.44**^**a**^	**86.89 ± 0.64**^**a**^	**49.65 ± 0.36**^**a**^
September	90.93 ± 0.67^b^	40.42 ± 0.30^b^	23.09 ± 0.17^b^

Values are expressed as mean ± SE (*n* = 3). Different superscript letters within the same column indicate statistically significant differences at p < 0.05 according to Duncan’s Multiple Range Test

### HPLC determination of phenolics and flavonoids

**Fig. 2:**
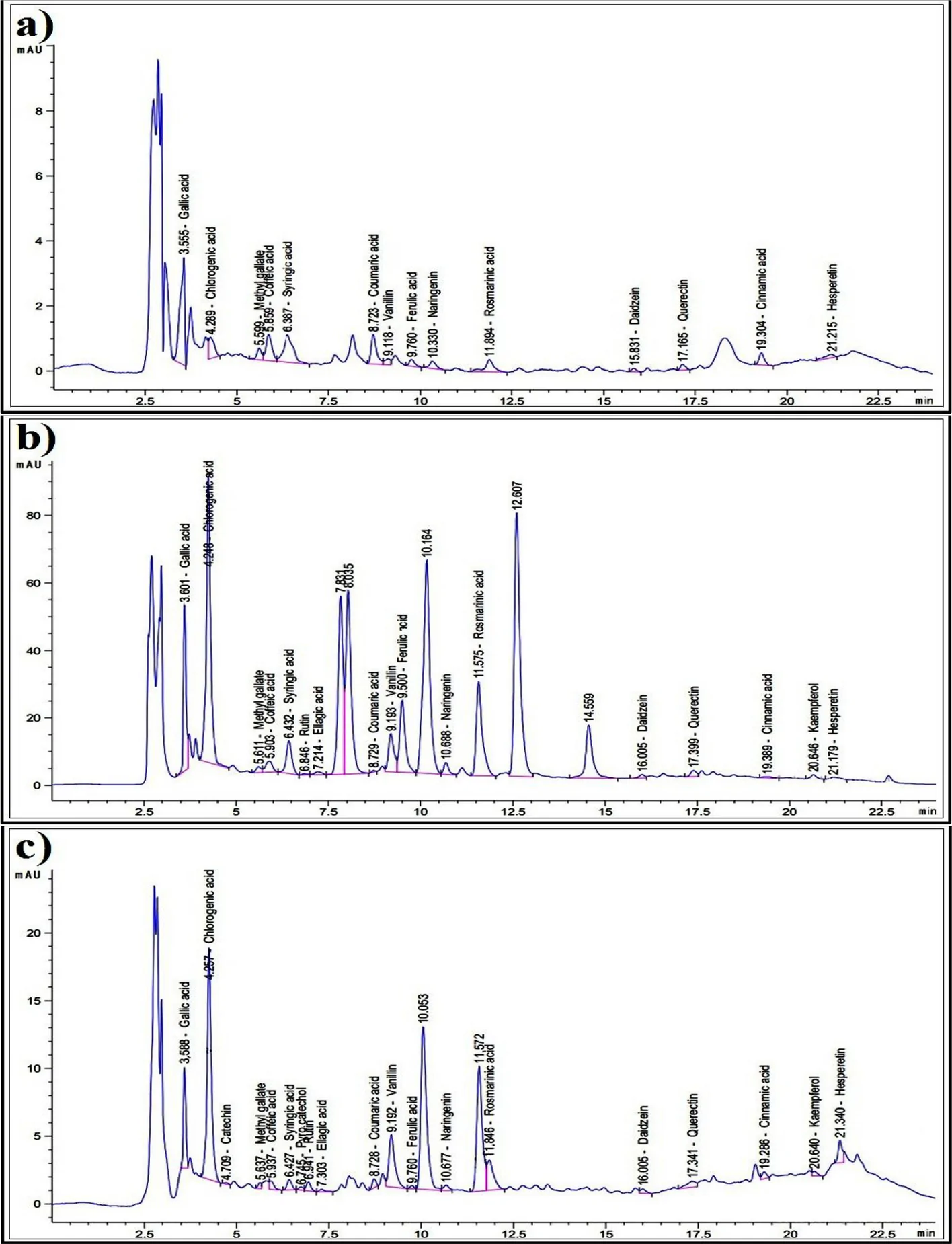
HPLC chromatogram of each extract from* V. vinifera* leaves harvested at **a)** March, **b)** May, and **c)** September

The aim of this evaluation was to assess the variation in polyphenolic compounds in *Vitis vinifera* leaves harvested at different periods. HPLC analysis revealed the presence of 19 phenolic and flavonoid compounds across all examined extracts. Specifically, 14 compounds were identified in the March extract, 17 compounds in the May extract, and 19 compounds in the September extract, as summarized in Table 3 and illustrated in Fig. 2.

**Table 3:** HPLC analysis for phenolic compounds and flavonoids identified in extract from *V. vinifera* leaves harvested at three different harvest times

No	Compound	March		May		September	
		**R**_**t**_** (min.)**	**Conc. (µg/g)**	**R**_**t**_** (min.)**	**Conc. (µg/g)**	**R**_**t**_** (min.)**	**Conc. (µg/g)**
1	Gallic acid	3.555	123.48	3.601	1097.85	3.588	154.58
2	Chlorogenic acid	4.289	46.74	4.248	4684.66	4.257	928.47
3	Catechin	0.00	–	0.00	–	10.77	4.709
4	Methyl gallate	5.599	8.21	5.611	41.24	5.637	6.40
5	Caffeic acid	5.859	37.11	5.903	176.06	5.937	21.35
6	Syringic acid	6.387	59.64	6.432	376.29	6.427	24.75
7	Pyrocatechol	0.00	–	0.00	–	6.745	14.00
8	Rutin	0.00	–	6.846	30.96	6.941	44.54
9	Ellagic acid	0.00	–	7.214	56.59	7.303	8.12
10	Coumaric acid	8.723	17.30	8.729	5.00	8.728	8.46
11	Vanillin	9.118	3.62	9.193	223.55	9.192	80.10
12	Ferulic acid	9.760	6.79	9.500	680.00	9.760	4.60
13	Naringenin	10.330	13.52	10.688	158.68	10.677	18.21
14	Rosmarinic acid	11.894	34.30	11.575	1748.48	11.846	151.81
15	Daidzein	15.831	2.60	16.005	30.66	16.005	9.86
16	Querectin	17.165	10.33	17.399	126.65	17.341	48.39
17	Cinnamic acid	19.304	3.53	19.389	6.76	19.286	4.08
18	Kaempferol	0.00	–	20.646	70.39	20.640	10.21
19	Hesperetin	21.215	5.25	21.179	59.28	21.340	34.53
**Total phenolic compounds concentration**		**340.72**		**9096.48**		**98.706**	
**Total flavonoids concentration**		**31.70**		**476.62**		**170.449**

The major phenolic acid and flavonoid constituents detected in the March and May leaf extracts were gallic and naringenin. In contrast, chlorogenic acid and were the predominant phenolic acid and flavonoid identified in the September extract. Catechin and pyrocatechol were absent from both the March and May extracts, while rutin, ellagic acid, and kaempferol were not detected in the March extract. Overall, the May-harvested leaves exhibited markedly higher concentrations of both phenolic and flavonoid compounds than those harvested in March and September, indicating that harvest timing significantly influences the accumulation of bioactive constituents in *V. vinifera* leaves.

### Characterization of the isolated compounds

Seven compounds were isolated from *V. vinifera* leaves extracts collected during March, May, and September using thin-layer chromatographic analysis (1–7). Structural elucidation was accomplished through the application of ESI–MS, ^1^H-NMR, and ^13^C-NMR techniques. The compounds quercetin (1), kaempferol (2), apigenin (3), apigenin-7-glucoside (4), and rutin (5) were identified by comparing their Co-TLC profiles with those of established authentic standards. The characterization process for compounds 6 and 7 was carried out as detailed below.

Compound 6 (*p*-coumaric acid**)**: Colorless needles (21 mg) with melting point 214 ͦ C. Rf 0.65. ESI–MS: *m/z* (relative abundance %): 164 (25) for molecular formula C_9_H_8_O_3_, 136(39), 120 (100), 107 (61), 91(75), and 65 (35). ^1^H-NMR (400 MHz, CD3OD-*d4*): δ ppm 6.98 (2H, *d,* J = 7.3, H-2 and H-6), 7.30 (2H, *d,* J = *7.3*, H-3 and H-5), 7.77(1H, *d*, J = 10.4, H-7), 6.28 (1H, *d*, J = 10.4, H-8). ^13^C-NMR (125 MHz, CD3OD-*d4*)**:** 165.17 (C-1), 119.12 (C-2, C-6), 133.18 (C-3, C-5), 126.12 (C-4), 147.44 (C-7), 117.47 (C- 8), 170.85 (C-9). The data are illustrated in Supplementary Figs. 1, 2, and 3. The isolated compound's spectral properties matched those published by [32].

Compound 7 (isorhamnetin, 3-methylquercetin): colorless powder (27.5 mg), with melting point 312 ͦ C, Rf 0.74, ESI–MS: m/z 316 (20) attributed to the molecular formula of C_16_H_12_O_7_, 301(27), 287 (38), 245 (61), 153 (72), 128 (53), 108 (64), 69(70). The ^1^H-NMR (400 MHz, CD3OD-*d4*): δ ppm 4.01 (3H, s, OCH3), 6.17 (1H, d, J = *3.2,* 6-H), 6.47 (1H, d, J = *3.2,* 8-H), 6.84 (1H, d, J = *4.1,* 5'-H), 7.59 (1H, m, 6'-H), 7.87 (1H, d, J = *4.1,* 2'-H). ^13^C-NMR (125 MHz, CD3OD-*d4*)**:**150.85 (C-2), 139.88 (C-3), 171.14 (C-4), 154.71 (C-5), 98.14 (C-6), 163.83(C-7), 90.81 (C-8), 159.31 (C-9), 103.57 (C-10), 125.85 (C-1'), 112.71 (C-2'), 140.18 (C-3'), 148.73 (C-4'), 114.73 (C-5'), 119.88 (C-6'), 55.57 (OCH_3_). The data are illustrated in Supplementary Figs. 4, 5, and 6. The results agreed with those reported by Aboul [1] in the literature.

In the examination of the three extracts from *V. vinifera* leaves, quercetin (1), apigenin (3), and apigenin-7-glucoside (4) were successfully identified. Conversely, Kaempferol (2) and rutin (5) were detected solely in the extracts from *V. vinifera* leaves gathered in May and September. Moreover, *p*-coumaric acid (Compound 6) and isorhamnetin (Compound 7) were identified only in the extract from *V. vinifera* leaves collected in May.

### In vitro biological activities

#### Antioxidant activity

The biological properties of these extracts, such as their anti-inflammatory, antidiabetic, anti-Alzheimer's, antioxidant, and radical scavenging properties, were evaluated and compared with the reference drug for each test. Therefore, the methanolic extract of May leaves had higher TAC and IRP (293.26 ± 2.15 mg gallic acid/g and 265.51 ± 2.15 µg/mL, respectively) compared to the March and September leaf extracts (78.20 ± 0.57 mg gallic acid/g and 50.45 ± 0.57 µg/mL, respectively) (136.40 ± 1.00 mg gallic acid/g and 108.65 ± 1.00 µg/mL, respectively). This suggests that the leaves harvested in May exhibited the most effective in vitro antioxidant activity, followed by those harvested in September and March **(**Table 4**)**.

**Table 4:** The in vitro antioxidant activity of extract from *V. vinifera* leaves harvested at three different times

**Harvest time**	**TAC** **(**mg gallic acid/g**)**	**IRP** **(**µg/mL**)**
March	78.20 ± 0.57^c^	50.45 ± 0.57^c^
May	**293.26 ± 2.15**^**a**^	**265.51 ± 2.15**^**a**^
September	136.40 ± 1.00^b^	108.65 ± 1.00^b^

Values are expressed as mean ± SE (*n* = 3). Different superscript letters within the same column indicate statistically significant differences at p < 0.05 according to Duncan’s Multiple Range Test

Values are expressed as mean ± SE (standard error) of three independent determinations (*n* = 3). Different superscript letters within the same column indicate significant differences at *p* < 0.05 according to Duncan's Multiple Range Test.

#### Scavenging activity

At equal concentration (100 µg/mL), the methanolic extract of May leaves demonstrated the highest inhibitory percentages (60.16 ± 0.44, 63.91 ± 0.44, and 54.41 ± 0.44%, respectively), indicating greater scavenging potential against DPPH, ABTS, and NO radicals, according to the data shown in Table 5. It was observed that the extracts with superior antioxidant effect had lesser IC_50_ values. As a result, when compared to the other extracts under study, the lowest IC_50_ values were 5.33 ± 0.02, 4.02 ± 0.03, and 6.44 ± 0.03 µg/mL, respectively. The leaves gathered in May showed the highest in vitro antioxidant activity, followed by those harvested in September and March, according to the antioxidant activity data.

**Table 5:** The in vitro radicals scavenging activities of extract from *V. vinifera* leaves harvested at three different times

Harvest time	DPPH		ABTS		NO	
	**Inhib** **(**%**)**	**IC**_**50**_ **(**µg/mL**)**	**Inhib** **(**%**)**	**IC**_**50**_ **(**µg/mL**)**	**Inhib** **(**%**)**	**IC**_**50**_ **(**µg/mL**)**
**March**	16.04 ± 0.12^d^	19.97 ± 0.09^a^	19.79 ± 0.12^d^	12.98 ± 0.08^a^	10.29 ± 0.12^d^	34.03 ± 0.26^a^
**May**	**60.16 ± 0.44**^**b**^	**5.33 ± 0.02**^**c**^	**63.91 ± 0.44**^**b**^	**4.02 ± 0.03**^**c**^	**54.41 ± 0.44**^**b**^	**6.44 ± 0.03**^**c**^
**September**	27.98 ± 0.21^c^	11.45 ± 0.05^b^	31.73 ± 0.21^c^	8.10 ± 0.06^b^	22.23 ± 0.21^c^	15.75 ± 0.09^b^
**Ascorbic Acid**	66.47 ± 0.14^a^	4.82 ± 0.02^d^	69.62 ± 0.14^a^	3.69 ± 0.01^d^	61.32 ± 0.14^a^	5.71 ± 0.02^d^

Values are expressed as mean ± SE (n = 3). Different superscript letters within the same column indicate statistically significant differences at *p* < 0.05 according to Duncan’s Multiple Range Test

The leaves gathered in March had the lowest scavenging activity (16.04 ± 0.12, 19.79 ± 0.12, and 10.29 ± 0.12%, respectively). As a result, this extract showed the highest IC_50_ values (19.97 ± 0.09, 12.98 ± 0.08, and 34.03 ± 0.26 µg/mL, respectively). However, at the same concentration, the ascorbic acid employed as a standard exhibited the lowest IC_50_ values (4.82 ± 0.02, 3.69 ± 0.01, and 5.71 ± 0.02 µg/mL, respectively) and the highest Inhib. % (66.47 ± 0.14, 69.62 ± 0.14, and 61.32 ± 0.14%, respectively).

#### Anti-diabetic activity

The effectiveness of the extract in combating diabetes is assessed by evaluating the inhibition of α-amylase and α-glucosidase, and this is compared to the efficacy of acarbose. It was evaluated against the activities of α-amylase and α-glucosidase enzymes in the current study. The methanolic extract of the May-harvested leaves had the lowest IC_50_ values (3.90 ± 0.03 and 3.05 ± 0.04 µg/mL, respectively) and a higher inhibitory effect against the activities of both enzymes (60.86 ± 0.42 and 50.61 ± 0.42%, respectively) at the same concentration (100 µg/mL). Following this extract were the leaves that were taken in September and March, respectively. At the same concentration, the leaves collected in March exhibited the highest IC_50_ values (12.60 ± 0.11 and 17.96 ± 0.30 µg/mL, respectively) and the lowest inhibitory impact on the activities of both enzymes (18.85 ± 0.11 and 8.60 ± 0.11%, respectively). In contrast, the strongest antidiabetic action was demonstrated by acarbose, which was used as a standard at the same concentration (69.11 ± 0.14 and 58.86 ± 0.14%, respectively). Out of all the extracts that were examined, acarbose had the lowest IC_50_ values (3.44 ± 0.03 and 2.62 ± 0.02 µg/mL, respectively) (Table 6).

**Table 6:** The in vitro antidiabetic and anti-Alzheimer activities of extract from *V. vinifera* leaves harvested at three different times

Harvest time	Antidiabetic Activity				Anti-Alzheimer Activity	
	**α-Amylase**		**α-Glucosidase**		**AChE**	
	**Inhib** **(**%**)**	**IC**_**50**_ **(**µg/mL**)**	**Inhib** **(**%**)**	**IC**_**50**_** (**µg/mL**)**	**Inhib** **(**%**)**	**IC**_**50**_ **(**µg/mL**)**
**March**	18.85 ± 0.11^d^	12.60 ± 0.11^a^	8.60 ± 0.11^d^	17.96 ± 0.30^a^	9.89 ± 0.13^d^	32.86 ± 0.43^a^
**May**	**60.86 ± 0.42**^**b**^	**3.90 ± 0.03**^**c**^	**50.61 ± 0.42**^**b**^	**3.05 ± 0.04**^**c**^	**58.21 ± 0.48**^**b**^	**5.58 ± 0.05**^**c**^
**September**	30.22 ± 0.20^c^	7.86 ± 0.07^b^	19.97 ± 0.20^c^	7.73 ± 0.11^b^	22.96 ± 0.23^c^	14.15 ± 0.14^b^
**STD**	**Acarbose**				**Donepezil**	
	**69.11 ± 0.14**^**a**^	**3.44 ± 0.03**^**d**^	**58.86 ± 0.14**^**a**^	**2.62 ± 0.02**^**d**^	**67.68 ± 0.16**^**a**^	**4.80 ± 0.02**^**d**^

Values are expressed as mean ± SE (*n* = 3). Different superscript letters within the same column indicate statistically significant differences at *p* < 0.05 according to Duncan’s Multiple Range Test

**Fig. 3:**
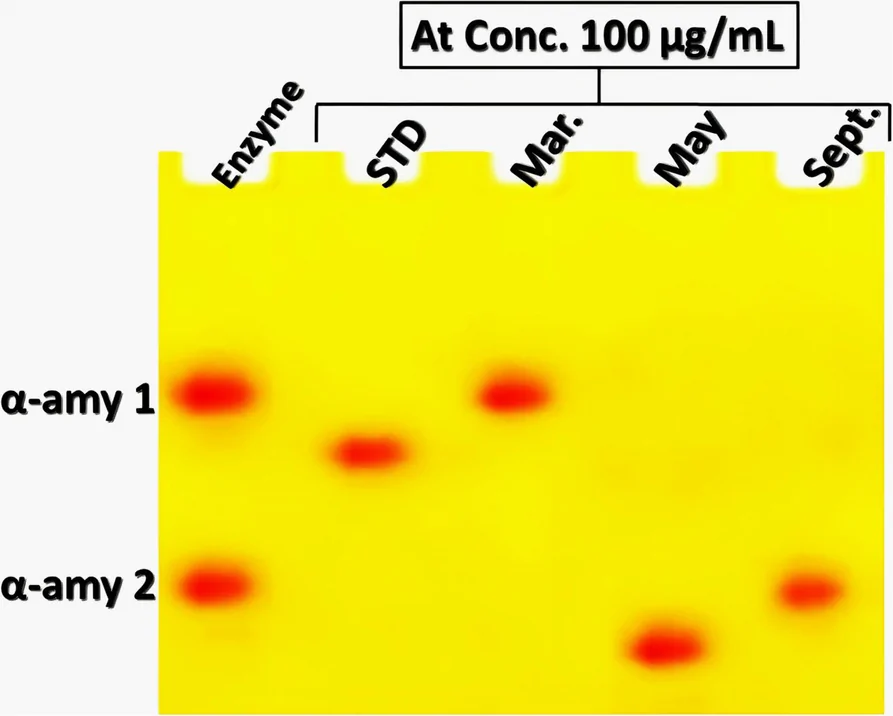
Native electrophoretic α-amylase isoenzyme pattern showing the antidiabetic activity of *V. vinifera* leaf extracts harvested in March, May, and September, compared to Acarbose (standard). Lanes were cropped from the same gel and are now marked with white dividing lines to indicate non-adjacent positions. Full-length, unprocessed gels are unavailable; cropped figures with annotations are provided in Supplementary Figs. 7 and 8

Various proteins and isoenzymes were isolated, recognized, and measured through electrophoresis, As presented in Fig. 3 and compiled in Supplementary Table 1, it was found that two distinct types of the typical α-amylase enzyme were electrophoretically represented at Rfs 0.28 and 0.55 (Qty 22.12 and 19.26; B% 53.46 and 46.54, respectively). 100 µg/mL of the extract from *V. vinifera* leaves that were gathered at three separate times were added to the standard enzyme. The standard (α-amylase) enzyme treated with extract from *V. vinifera* leaves collected in March showed qualitative anomalies in the electrophoretic isoenzyme pattern, indicated by the loss of one typical type (α-amy 2). Consequently, the electrophoretic α-amylase patterns of this treated sample are physiologically equivalent to the reference enzyme 66.67% of the time (Diff. = 33.33%).

When the extract from *V. vinifera* leaves collected in May was added to the standard enzyme, the electrophoretic isoenzyme pattern was altered. The appearance of one aberrant band at Rf 0.64 (Qty 21.88 and B.% 100.00) and the disappearance of two normal bands provided evidence for this. The electrophoretic α-amylase pattern of this sample is therefore completely different from that of the reference enzyme (SI = 0.00%; Diff. = 100.00%) in the physiological state. One normal type (α-amy 1) disappearing is thought to be an indication that the standard enzyme has been treated with an extract from September-collected *V. vinifera* leaves. The electrophoretic α-amylase pattern of this sample is therefore physiologically equivalent to that of the reference enzyme in 66.67% of cases (Diff. = 33.33%). Supplementary Fig. 7. displays the unprocessed α-amylase isoenzyme pattern. Supplementary Fig. 8. displays the unprocessed α-amylase isoenzyme pattern (analyzed image).

**Fig. 4:**
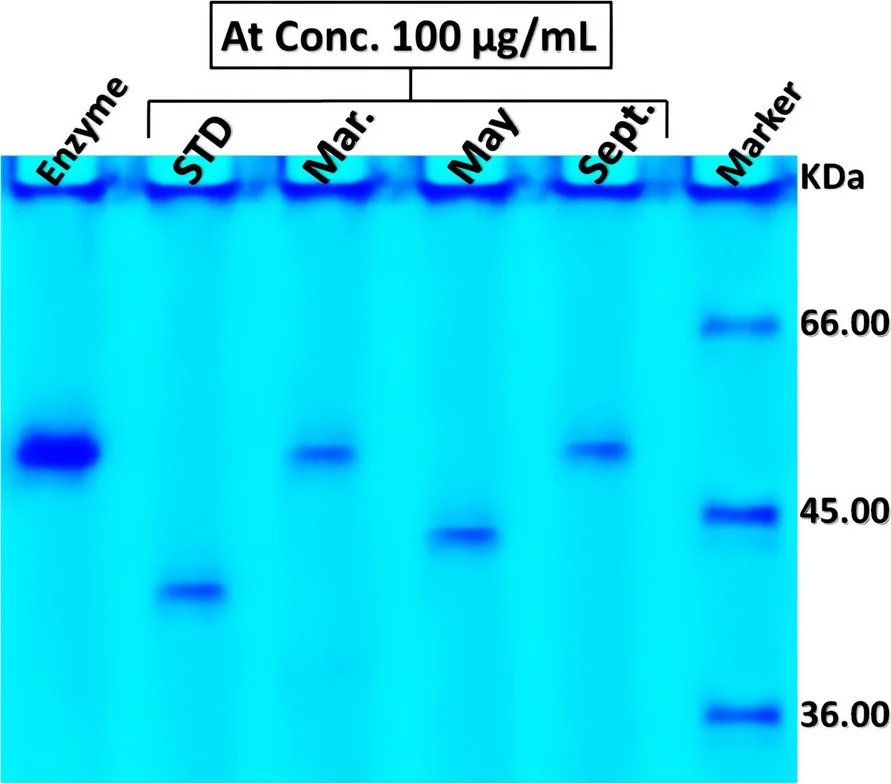
SDS-PAGE pattern showing the effect of *V. vinifera* leaf extracts on α-glucosidase enzyme levels, compared to Acarbose. Cropped lanes are separated by visible white space. Full-length gels are unavailable; figures with annotations are provided in Supplementary Figs. 9 and 10

As shown in Fig. 4 and Supplementary Table 2, the α-glucosidase was detected and verified using SDS-PAGE as a single band with a molecular weight of 48 kDa, in agreement with the work carried out by [35]. In the present study, the crude α-glucosidase enzyme was identified as a single band at Rf 0.40 (Qty 51.08 and B% 20.47). The extracts from *V. vinifera* leaves gathered at three different periods were identified electrophoretically at equal concentrations (100 µg/mL), and SDS-PAGE was used to display the quantitative differences between them. The extract obtained in May had the highest denaturing action against α-glucosidase, as demonstrated by a 50.19% (Qty 10.20) drop in the amount of the detected band as the molecular weight decreased from 51.08 to 43.99 kDa. Supplementary Fig. 9. displays unprocessed α-glucosidase isoenzyme pattern. Supplementary Fig. 10. displays unprocessed α-glucosidase isoenzyme pattern (Analyzed Image). When the standard enzyme was treated with the September extract, the amount of the band decreased by 30.35% (Qty 14.26) without changing the molecular weight, impairing the α-glucosidase. The extract extracted in March had the least degree of antidiabetic effect, lowering the band's quantity by 15.64% (Qty 17.27) without changing the molecular weight. Acarbose, a typical drug, completely denatured the electrophoretic isoenzyme pattern, as evidenced by a 72.29% decrease in the amount of the band (Qty 5.67), as the molecular weight decreased from 51.08 to 41.36 kDa.

#### Anti-Alzheimer's activity

The methanolic extract of the May-harvested leaves exhibited a stronger inhibitory effect on the AChE enzyme (58.21 ± 0.48%) than the other extracts under study at the same concentration (100 µg/mL), according to the findings in Table 6. Consequently, the extract of the leaves that were obtained in September (Inhib. 22.96 ± 0.23%; IC_50_ 14.15 ± 0.14 µg/mL) and March (Inhib. 9.89 ± 0.13%; IC_50_ 32.86 ± 0.43 µg/mL) was found to have the lower IC_50_ value (5.58 ± 0.05 µg/mL). In contrast to the other examined extracts, donepezil, which was utilized as a standard at the same concentration, demonstrated the strongest inhibitory effect on the AChE enzyme (67.68 ± 0.16%) and the lowest IC_50_ value (4.80 ± 0.02 µg/mL).

#### Anti-arthritic activity

Compared to the other tested extracts at the same concentration (100 µg/mL), the methanolic extract of the May-harvested leaves showed a stronger inhibitory effect on protein denaturation (48.69 ± 0.34%) and the activity of the proteinase enzyme (Inhib. 45.54 ± 0.34%; IC_50_ 7.74 ± 0.06 µg/mL). The protein denaturation (15.08 ± 0.09%) and proteinase enzyme activity (Inhib. 11.93 ± 0.09%; IC_50_ 29.55 ± 0.22 µg/mL) were both most inhibited by the extract of the March-harvested leaves. Comparing all of the tested extracts, diclofenac sodium, which was used as a standard at the same dosage, had the strongest inhibitory impact on protein denaturation (55.28 ± 0.11%) and proteinase enzyme activity (Inhib. 52.13 ± 0.11%; IC_50_ 6.76 ± 0.03 µg/mL) **(**Table 7**)**.

**Table 7:** The in vitro anti-arthritic activity of extract from *V. vinifera* leaves harvested at three different times

Harvest time	Protein Denaturation	Proteinase	
	**Inhib** **(**%**)**	**Inhib** **(**%**)**	**IC**_**50**_ **(**µg/mL**)**
**March**	15.08 ± 0.09^a^	11.93 ± 0.09^d^	29.55 ± 0.22^a^
**May**	**48.69 ± 0.34**^**b**^	**45.54 ± 0.34**^**b**^	**7.74 ± 0.06**^**c**^
**September**	24.18 ± 0.16^c^	21.03 ± 0.16^c^	16.76 ± 0.12^b^
**Diclofenac Sodium**	**55.28 ± 0.11**^**a**^	**52.13 ± 0.11**^**a**^	**6.76 ± 0.03**^**d**^

Values are expressed as mean ± SE (n = 3). Different superscript letters within the same column indicate statistically significant differences at p < 0.05 according to Duncan’s Multiple Range Test

#### Anti-inflammatory activity

The methanolic extract of the May-harvested leaves exhibited a greater inhibitory effect on the activities of COX-1, COX-2, and 5-LOX enzymes (61.48 ± 0.45, 63.73 ± 0.45, and 54.77 ± 0.14%, respectively) compared to the other examined extracts at the same dose (100 µg/mL). Therefore, lower IC_50_ values (IC_50_ 6.15 ± 0.03, 4.68 ± 0.02, and 7.42 ± 0.02 µg/mL, respectively) were observed for this extract. The methanolic extract of the September-harvested leaves (with a lesser inhibitory effect of 16.10 ± 0.12, 18.35 ± 0.12, and 11.10 ± 0.12%, respectively) was next, followed by the March-harvested leaves. Therefore, higher IC_50_ values (IC_50_ 23.47 ± 0.10, 16.27 ± 0.05, and 36.62 ± 0.41 µg/mL, respectively) were observed for this extract. In contrast, the highest inhibitory activity against the activities of the COX-1 and COX-2 enzymes was demonstrated by indomethacin, which was used as a standard at the same dose (67.77 ± 0.14 and 70.02 ± 0.14%, respectively). The 5-LOX enzyme's activity was measured using zileuton as a reference, and it showed the strongest inhibitory impact (56.48 ± 0.45%). When compared to all the extracts under study, indomethacin (5.58 ± 0.02 and 4.26 ± 0.02 µg/mL) and zileuton (7.20 ± 0.06 µg/mL) had the lowest IC_50_ values **(**Table 8**)**.

**Table 8:** The in vitro anti-inflammatory activity of extract from *V. vinifera* leaves harvested at three different times

Harvest time	COX-1		COX-2		5-LOX	
	**Inhib** **(**%**)**	**IC**_**50**_ **(**µg/mL**)**	**Inhib** **(**%**)**	**IC**_**50**_ **(**µg/mL**)**	**Inhib** **(**%**)**	**IC**_**50**_ **(**µg/mL**)**
**March**	16.10 ± 0.12^d^	23.47 ± 0.10^a^	18.35 ± 0.12^d^	16.27 ± 0.05^a^	11.10 ± 0.12^d^	36.62 ± 0.41^a^
**May**	**61.48 ± 0.45**^**b**^	**6.15 ± 0.03**^**c**^	**63.73 ± 0.45**^**b**^	**4.68 ± 0.02**^**c**^	**54.77 ± 0.14**^**b**^	**7.42 ± 0.02**^**c**^
**September**	28.38 ± 0.21^c^	13.32 ± 0.06^b^	30.63 ± 0.21^c^	9.75 ± 0.03^b^	23.38 ± 0.21^c^	17.39 ± 0.16^b^
**STD**	**Indomethacin**				**Zileuton**	
	**67.77 ± 0.14**^**a**^	**5.58 ± 0.02**^**d**^	**70.02 ± 0.14**^**a**^	**4.26 ± 0.02**^**d**^	**56.48 ± 0.45**^**a**^	**7.20 ± 0.06**^**d**^

Values are expressed as mean ± SE (*n* = 3). Different superscript letters within the same column indicate statistically significant differences at *p* < 0.05 according to Duncan’s Multiple Range Test

#### Cytotoxic activity and enzymatic assays

**Fig. 5:**
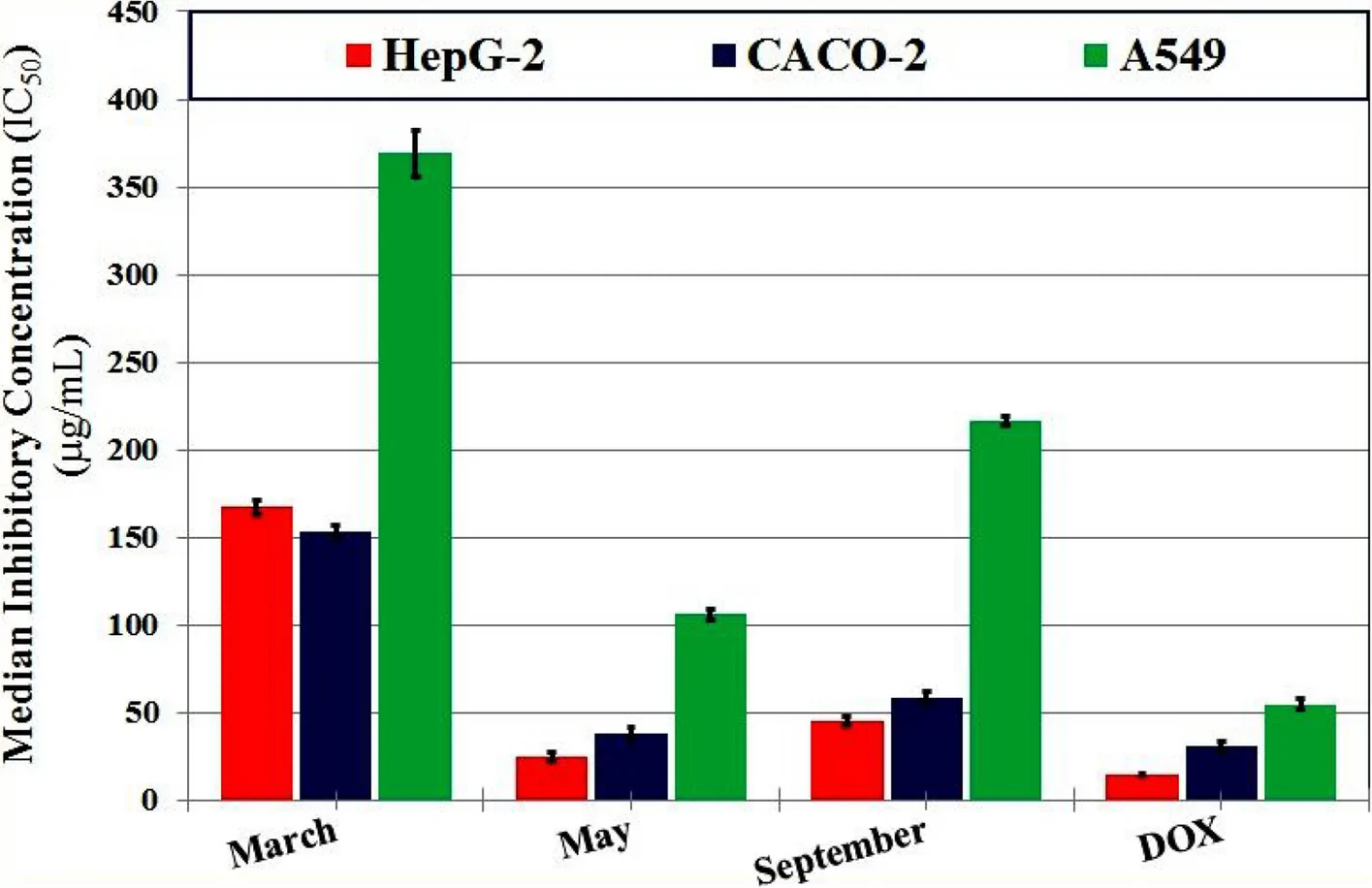
The in vitro cytotoxic activities of extract from *V. vinifera* leaves harvested at three different times against human hepatocellular carcinoma (HepG-2), colon (CACO-2) and lung (A549) cancer cell lines. The data was calculated from n = 3/extract and shown as mean ± SE

The methanolic extract of the May-harvested leaves demonstrated the uppermost cytotoxic effect against HepG-2, CACO-2, and A549 cells with less IC_50_ value (IC_50_ 24.53 ± 2.62, 37.73 ± 3.57, and 105.82 ± 3.53 μg/mL, respectively). The leaves taken in March were followed by the methanolic extract of the September leaves, which had higher IC_50_ values of 45.21 ± 2.58, 57.90 ± 3.60, and 216.31 ± 2.55 μg/mL, respectively. As a result, this extract showed higher IC_50_ values (IC_50_ 167.00 ± 3.79, 153.26 ± 3.57, and 368.97 ± 13.10 µg/mL, respectively). Of all the extracts that were examined, doxorubicin, which was used as a standard, had the highest cytotoxic activity (IC_50_ 14.38 ± 0.86, 30.70 ± 2.65, and 54.79 ± 2.86 μg/mL, respectively) (Fig. 5).

The percentages of viability and cytotoxicity at various concentrations of extract derived from *V. vinifera* leaves, collected at three distinct harvest times, against HEPG-2, CACO-2, and A549 cells are detailed in Supplementary Tables 3, 4, and 5, along with the calculated IC_50_ values for in vitro cytotoxic activity presented in Supplementary Table 6. The methanolic extract of May-harvested leaves exhibited the lowest IC_50_ values and the highest cytotoxic activity against the three cell lines studied (HepG-2, CACO-2, and A549 cells).

**Fig. 6:**
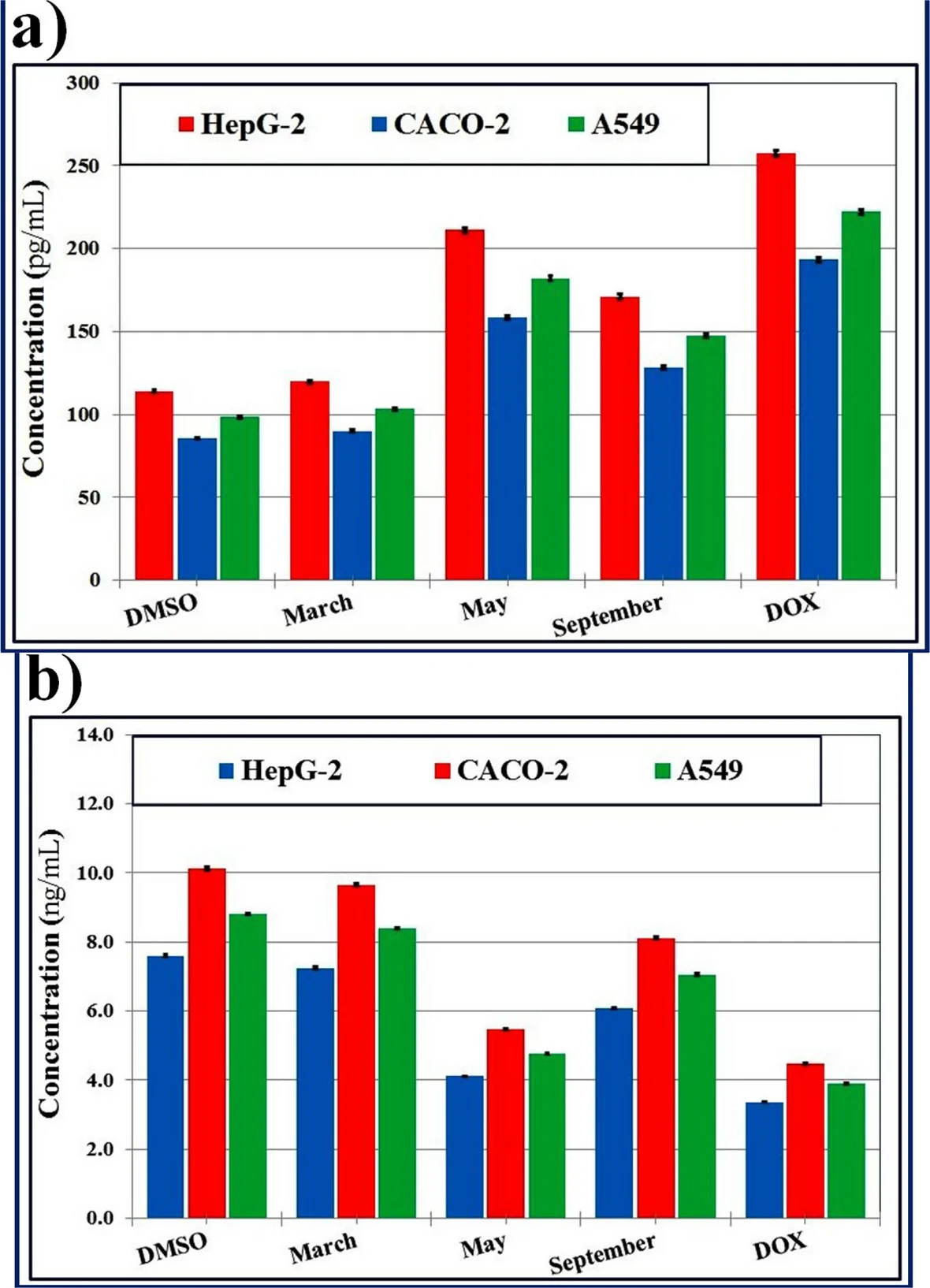
Data of the enzymatic assay showing the effect of extract from *V. vinifera* leaves harvested at three different times on **a)** Caspase-3 and **b)** Bcl-2 of human hepatocellular carcinoma (HepG-2), colon (CACO-2) and lung (A549) cancer cells

Following treatment of the HepG-2, CACO-2, and A549 cells with the various extracts of *V. vinifera* leaves under study, the activities of the caspase-3 and Bcl-2 enzymes were measured in the treated cells at IC_50_ values. Supplementary Table 7 illustrate the enzymatic assay values after the treatment of human hepatocellular carcinoma (HepG-2), colon (CACO-2) and lung (A549) cancer cells with of extract from *V. vinifera* leaves harvested at three different times. The highest activity of the caspase-3 enzyme was observed in HepG-2 (211.14 ± 1.41 pg/mL), CACO-2 (158.36 ± 1.06 pg/mL), and A549 (182.11 ± 1.22 pg/mL) treated with the methanolic extract of May-harvested leaves, with the lowest activity of the Bcl-2 enzyme (4.11 ± 0.01, 5.48 ± 0.02, and 4.76 ± 0.02 ng/mL, respectively) observed in these treated cells (Fig. 6).

The HepG-2, CACO-2, and A549 cells treated with the methanolic extract of March-harvested leaves showed the highest activity of the Bcl-2 enzyme (7.60 ± 0.03, 10.13 ± 0.04, and 8.81 ± 0.03 ng/mL, respectively), while the caspase-3 enzyme activity was lower (114.13 ± 0.76, 85.60 ± 0.57, and 98.44 ± 0.66 pg/mL, respectively). When HepG-2, CACO-2, and A549 cells were treated with doxorubicin, the highest activity of the caspase-3 enzyme (257.65 ± 1.72, 193.24 ± 1.29, and 222.22 ± 1.49 pg/mL, respectively) and the lowest activity of the Bcl-2 enzyme (3.37 ± 0.01, 4.49 ± 0.02, and 3.90 ± 0.01 pg/mL, respectively) were observed.

## Discussion

The present study demonstrated clear harvest-dependent changes in the pigment composition, phytochemical profile, and in vitro biological activities of *Vitis vinifera* leaves. Overall, the May harvest showed the most pronounced accumulation of chlorophylls and major phenolic constituents and consistently exhibited the strongest biological activities, whereas March generally showed the lowest phytochemical content and biological activity. These findings indicate that harvest timing is an important factor affecting the functional quality of grapevine leaves.

Photosynthetic pigments provide useful indicators of leaf physiological status and developmental changes. Chlorophylls are central to light absorption and photosynthetic energy conversion, whereas carotenoids participate in light harvesting and photoprotection by transferring energy to chlorophyll and protecting the photosynthetic apparatus from oxidative damage [43, 45]. Consequently, changes in pigment concentrations can reflect developmental and physiological transitions during the leaf life cycle [79, 88]. In the present study, chlorophyll *a*, chlorophyll *b*, and total chlorophylls were significantly higher in May than in March and September, while carotenoids showed the opposite pattern, reaching their highest concentration in March and decreasing markedly in May before increasing again in September. The high chlorophyll content during May is consistent with active vegetative growth and a fully functional photosynthetic apparatus. Conversely, the decline in chlorophyll toward September is consistent with the physiological changes accompanying late-season maturation and the onset of senescence. Seasonal variation in leaf pigment content has previously been reported in grapevine and other plants [77]. Our findings are also comparable with the observations of [75], who reported changes in grape-leaf chlorophyll *a* during grape development, with an increase during ripening followed by a decline. [36] similarly observed substantial reductions in chlorophyll *a* and *b* from grape ripening to leaf fall. The relatively high carotenoid level observed in the present study during March may reflect the protective role of these pigments during early leaf development, while the subsequent changes in carotenoids may accompany alterations in chlorophyll metabolism. [89] reported that carotenoid degradation begins after chlorophyll degradation, supporting the temporal differences between the two pigment classes observed during leaf development.

The significant differences in total polyphenols, flavonoids, and tannins among the three harvest periods provide further evidence that the accumulation of secondary metabolites is strongly dependent on harvest timing. The May extract contained substantially higher levels of all three phytochemical groups than the March and September extracts, while March showed the lowest concentrations. Similar variation in the phytochemical composition of grape leaves according to harvest time has been reported previously [22, 74]. The accumulation of phenolic compounds in plants is influenced by multiple interacting factors, including genotype, maturity, geographical conditions, temperature, light intensity, water availability, agricultural practices, and harvest time [23, 42]. [48] also emphasized the influence of climatic, genetic, and agronomic factors on phenolic accumulation. Thus, the higher phenolic and flavonoid contents observed in May may reflect the physiological status of the leaves during active vegetative growth and the associated metabolic demand for protective secondary metabolites.

The present findings are consistent with [22], who demonstrated that grape leaves are important sources of phenolic and flavonoid compounds but that their concentrations vary according to harvest period. The results also agree with [48], who demonstrated that environmental and agronomic factors can substantially affect the phenolic composition of grape leaves. Collectively, these observations emphasize that harvest time should be considered when selecting grapevine leaves as a source of bioactive phytochemicals.

The HPLC analysis further demonstrated that harvest time affected not only the total amount of phenolics but also the composition of individual constituents. Nineteen phenolic and flavonoid compounds were detected across the three harvest periods, with 14 compounds detected in March, 17 in May, and 19 in September. However, the May extract showed a markedly greater overall concentration of the identified compounds, particularly chlorogenic acid, gallic acid, rosmarinic acid, and naringenin. Chlorogenic acid reached 4684.66 μg/g extract and gallic acid 1097.85 μg/g extract in the May sample.The detection of hydroxycinnamic acids, phenolic acids, and flavonoids in grape leaves agrees with previous reports describing grapevine leaves as important sources of these compounds [22, 51]. The pronounced increase in several constituents during May is also consistent with [37] and [83], who reported significant seasonal and developmental variation in grape-leaf phenolic metabolites.

The observed changes may reflect differences in secondary metabolism during leaf development. Active vegetative growth is associated with substantial metabolic activity, and phenylpropanoid-derived compounds can accumulate as protective metabolites in response to environmental and oxidative stresses [18, 23]. Conversely, reductions in individual metabolites during later development may result from their transformation, incorporation into structural components, degradation, or changes in their biosynthetic pathways during maturation and senescence [77, 86]. Environmental factors such as temperature, light intensity, and water availability may further regulate enzymes involved in phenolic biosynthesis [23, 48]. The greater abundance of phenolic and flavonoid constituents in May is particularly relevant to the biological findings of the present study. Phenolic compounds possess diverse structural features that influence their antioxidant and other biological activities. The biological properties of flavonoids are related to their C6–C3–C6 skeleton and the degree and position of hydroxylation and methoxylation [5, 27, 28]. Thus, the harvest-dependent differences in the qualitative and quantitative composition of these compounds provide a plausible explanation for the differences in biological activity observed among the extracts.

The May extract demonstrated the highest total antioxidant capacity and iron-reducing power, followed by the September and March extracts. The same pattern was observed in the DPPH, ABTS, and nitric oxide radical-scavenging assays, in which the May extract exhibited the highest inhibition and the lowest IC₅₀ values. This parallel between phytochemical accumulation and antioxidant activity is consistent with the recognized contribution of phenolic compounds and flavonoids to antioxidant capacity [64, 82]. The present findings agree with [22], who reported substantial radical-scavenging activity in grape-leaf extracts associated with their phenolic and flavonoid content. [42] likewise demonstrated that variation in grape-leaf phytochemical composition can influence antioxidant capacity. The stronger activity of the May extract may therefore reflect its higher concentration of chlorogenic acid, gallic acid, rosmarinic acid, flavonoids, and tannins. However, because the present study did not experimentally establish individual compound–activity relationships, the enhanced antioxidant activity should be interpreted as being associated with the overall phytochemical enrichment rather than attributed to a single compound.

The May extract also showed the strongest inhibition of both α-amylase and α-glucosidase, with lower IC₅₀ values than the March and September extracts, although acarbose remained more active. These findings indicate that the harvest period influences the carbohydrate-hydrolyzing enzyme inhibitory potential of grape-leaf extracts. The stronger activity of the May extract coincided with its greater abundance of phenolic and flavonoid constituents. Phenolic structures and the number and orientation of their functional groups have been proposed to influence inhibition of carbohydrate-hydrolyzing enzymes [58]. Polyphenolic compounds may interact directly with enzyme active sites and interfere with substrate hydrolysis, providing a possible explanation for the greater inhibition observed in the phytochemically richer May extract [47, 49]. The electrophoretic experiments provided additional evidence of harvest-dependent effects on enzyme-associated patterns. Treatment with the different extracts altered the α-amylase and α-glucosidase electrophoretic profiles to different extents, with the May extract producing the most pronounced changes. Alterations in electrophoretic band patterns may reflect changes in protein structure or enzyme-associated characteristics [63, 87]. The observed changes in α-glucosidase patterns may also be related to interactions between phenolic constituents and the enzyme, potentially affecting its structural integrity and activity [38, 53].

The May extract exhibited the strongest AChE inhibitory activity among the three harvest periods, although its activity remained slightly lower than that of donepezil. The enhanced AChE inhibition observed for the May extract is consistent with its greater phytochemical enrichment. Phenolic compounds can interact with amino acid residues within the AChE active site through hydrogen bonding and hydrophobic interactions, while compounds containing multiple hydroxyl groups may exhibit stronger binding interactions [3]. The antioxidant properties of grape-leaf constituents may also contribute to their neuroprotective potential. Nevertheless, the present results represent an in vitro enzyme-inhibition finding and should not be interpreted as evidence of anti-Alzheimer therapeutic efficacy. Rather, they indicate that the May-harvested extract warrants further investigation as a source of compounds with AChE inhibitory potential.

The May extract exhibited the greatest inhibition of protein denaturation and proteinase activity among the investigated extracts, although diclofenac sodium remained more potent. The higher activity of the May extract may be related to its greater content of phenolic and flavonoid constituents. Phenolic hydroxyl groups can participate in hydrogen bonding and other interactions with proteins, potentially affecting proteinase activity [19, 71, 81]. The reported presence of proanthocyanidins in grape leaves may also contribute to antioxidant and anti-inflammatory properties [21]. Similarly, the May extract showed the strongest inhibition of COX-1, COX-2, and 5-LOX. These enzymes are important mediators of inflammatory pathways: COX enzymes participate in prostaglandin formation from arachidonic acid, while 5-LOX contributes to leukotriene and lipid-hydroperoxide production [50, 54, 57]. The greater inhibition observed for the May extract may therefore be associated with its enriched polyphenolic profile. Chlorogenic acid, rosmarinic acid, rutin, luteolin, and isoquercitrin have previously been associated with anti-inflammatory effects [8, 68], while structural characteristics of flavonoids, including hydroxylation and conjugation, may influence their biological activities [26].

Among the three harvest periods, the May extract exhibited the strongest cytotoxic activity against HepG-2, CACO-2, and A549 cells, as indicated by its lowest IC₅₀ values. This pattern is consistent with the greater accumulation of phenolic and flavonoid constituents detected in the May extract and with previous evidence that the chemical composition of plant extracts can influence their cytotoxic properties [60]. Flavonoids and tannins have been reported to exert antiproliferative effects through several mechanisms, including modulation of apoptosis, cell-cycle progression, and DNA-associated processes [6, 29, 31]. In the present study, treatment with the May extract increased caspase-3 activity and decreased Bcl-2 levels in all three tested cell lines. This pattern is consistent with activation of apoptosis and agrees with previous reports linking plant-derived bioactive constituents to increased caspase-3 activity and reduced expression of anti-apoptotic Bcl-2 [4, 59, 78, 85]. The simultaneous increase in caspase-3 and reduction in Bcl-2 observed for the May extract therefore provide preliminary evidence that apoptosis-related mechanisms may contribute to its cytotoxic activity. Nevertheless, these findings should be considered mechanistic indications rather than definitive proof of a specific apoptotic pathway**,** and further molecular studies would be required to establish the underlying mechanisms.

Taken together, the results demonstrate a consistent relationship between harvest time, phytochemical accumulation, and in vitro biological performance. The May harvest was characterized by the highest chlorophyll content, total polyphenols, flavonoids, and tannins, as well as enhanced levels of several individual phenolic constituents. This phytochemical profile was accompanied by the strongest antioxidant, enzyme-inhibitory, anti-inflammatory, and cytotoxic activities. Such consistency across chemically and biologically distinct assays supports harvest time as an important consideration in the selection of grapevine leaves as a source of bioactive material. The findings also provide a preliminary basis for the valorization of grapevine leaves as an underutilized agricultural by-product. However, the present study establishes biological potential rather than direct commercial applicability**.** Further work involving extract standardization, optimization of extraction conditions, compound–activity relationships, safety and toxicity assessment, stability studies, formulation, and economic evaluation would be necessary before industrial or commercial development can be proposed. Within this context, identification of May as the most phytochemically enriched harvest period provides a useful starting point for future development of standardized grape-leaf extracts.

A limitation of the present study is that the phytochemical characterization was primarily focused on targeted phenolic and flavonoid constituents using HPLC–DAD; therefore, comprehensive profiling of other metabolite classes was not performed. Future studies will employ complementary advanced analytical platforms, such as LC–MS/MS, GC–MS, and HPTLC, to provide a broader characterization of the harvest-dependent phytochemical variation in *Vitis vinifera* leaves.

## Conclusion

The present study demonstrated that the composition and concentration of phyto-constituents in *Vitis vinifera* leaves vary significantly with the harvest season. Among the examined periods, leaves collected in May exhibited the highest levels of phytochemicals and the most pronounced in vitro biological activities. In contrast, leaves harvested in September and March displayed comparatively lower values. These findings highlight the critical influence of harvest time on the phytochemical profile and associated biological effects of *V. vinifera* leaves, emphasizing the necessity of considering seasonal variation when collecting leaf samples for research or therapeutic applications.

## Supplementary Information

Below is the link to the electronic supplementary material.

Supplementary Material 1

## Data Availability

The manuscript has associated data as supplementary materials.

## References

[1] Aboul Naser A, Younis E, El-Feky A, Elbatanony M, Hamed M. Management of *Citrus sinensis* peels for protection and treatment against gastric ulcer induced by ethanol in rats. Biomarkers. 2020;25(4):349–59. 10.1080/1354750X.2020.1759693 32319821

[2] Aboul Naser AF, El-Feky AM, Hamed MA. Mitigating effect of *Lepidium sativum* seeds oil on ovarian oxidative stress, DNA abnormality and hormonal disturbances induced by acrylamide in rats. Chem Biodivers. 2024;21(7):e202400062. 10.1002/cbdv.202400062 38743868

[3] Aboulthana WM, El-Feky AM, Ibrahim NE, Soliman AAF, Youssef AM. Phytochemical analysis and biological study on Sinapis alba L. seeds extract incorporated with metal nanoparticles, in vitro approach Sci Rep. 2025; 15: 13782, P: 1-22 10.1038/s41598-025-95347-6. PMC12012182 40258907

[4] Aboulthana WM, Omar NI, El-Feky AM, Hassan AK, Hasan EA, Seif M, Youssef AM. Phyto- and biochemical study on cape gooseberry (*Physalis peruviana* L.) extract incorporated with metal nanoparticles against hepatic injury induced in rats. Natural Product Research. 2024: 1–14 (10.1080/14786419.2024.2358392). 38795161

[5] Ahmed YR, Aboul Naser AF, Elbatanony MM, El-Feky AM, Khalil WK, Hamed MA. Gene expression, oxidative stress, and neurotransmitters in rotenone-induced parkinson’s disease in rats: role of naringin from *Citrus aurantium* via blocking adenosine A2A receptor. Curr Bioact Compd. 2024;20(5):1–6.

[6] Al Qaisi Y, Alfarrayeh I, Alsarayreh A, Khleifat K, Abu-Nwas N. Assessment of antioxidant potential, cytotoxicity, and anticancer activity of methanolic extracts from selected wild medicinal plants. Phytomedicine Plus. 2024;4(2):100534.

[7] Alaa AM, El-Azab AS, Abou-Zeid LA, ElTahir KE, Abdel-Aziz NI, Ayyad RR, et al. Synthesis, anti-inflammatory, analgesic and COX-1/2 inhibition activities of anilides based on 5, 5-diphenylimidazolidine-2, 4-dione scaffold: molecular docking studies. Eur J Med Chem. 2016;115:121–31. 10.1016/j.ejmech.2016.03.011 26999325

[8] Ali DE, Gedaily RAE, Ezzat SM, El Sawy MA, Meselhy MR, Abdel-Sattar E. In silico and in vitro anti-inflammatory study of phenolic compounds isolated from *Eucalyptus maculata* resin. Sci Rep. 2023;13:2093. 10.1038/s41598-023-28221-y PMC9902548 36747067

[9] Amen Y, Sherif AE, Shawky NM, Abdelrahman RS, Wink M, Sobeh M. Grape-leaf extract attenuates alcohol-induced liver injury via interference with NF-κB signaling pathway. Biomolecules. 2020;10(4):558. 10.3390/biom10040558 PMC7225955 32268521

[10] Arnao MB, Cano A, Acosta M. The hydrophilic and lipophilic contribution to total antioxidant activity. Food Chem. 2001;73:239–44.

[11] Arora S, Itankar P. Extraction, isolation and identification of flavonoid from *Chenopodium album* aerial parts. J Tradit Complement Med. 2018;8(4):476–82. 10.1016/j.jtcme.2017.10.002 PMC6174260 30302328

[12] Arvouet-Grand A, Vennat B, Pourrat A, Legret P. Standardization of propolis extract and identification of principal constituents. J Pharm Belg. 1994;49(6):462–8. 7884635

[13] Bodnar OI, Viniarska HB, Vasilenko OV, Grubinko VV. Pigment content of Chlorella vulgaris Beij. under influence of sodium selenite and metals ions. Biotechnologia Acta. 2016;9(1):71–8.

[14] Borai IH, Ezz MK, Rizk MZ, Aly HF, El-Sherbiny M, Matloub AA, et al. Therapeutic impact of grape leaves polyphenols on certain biochemical and neurological markers in AlCl_3_-induced Alzheimer’s disease. Biomed Pharmacother. 2017;93:837–51. 10.1016/j.biopha.2017.07.038 28715867

[15] Bouderias S, Teszlák P, Jakab G, Kőrösi L. Age-and season-dependent pattern of flavonol glycosides in Cabernet Sauvignon grapevine leaves. Sci Rep. 2020;10(1):14241. 10.1038/s41598-020-70706-7 PMC7455724 32859977

[16] Broadhurst RB, Jones WT. Analysis of condensed tannins using acidified vanillin. J Sci Food Agric. 1978;48(3):788–94.

[17] Chakraborthy GS. Free radical scavenging activity of *Costus speciosus* leaves. Indian J Pharm Educ Res. 2009;43:96–8.

[18] Chen H, Yang J, Deng X, Lei Y, Xie S, Guo S, et al. Foliar-sprayed manganese sulfate improves flavonoid content in grape berry skin of Cabernet Sauvignon (*Vitis vinifera* L.) growing on alkaline soil and wine chromatic characteristics. Food Chem. 2020;314:126182. 10.1016/j.foodchem.2020.126182 31968293

[19] Chu Q, Bao B, Wu W. Mechanism of interaction between phenolic compounds and proteins based on non-covalent and covalent interactions. Med Res. 2018;2:180014.

[20] Das S, Sureshkumar P. Effect of methanolic root extract of *Blepharispermum subsesssile* DC in controlling arthritic activity. Res J Biotechnol. 2016;11(4):65–74.

[21] Deeban A, Anand K, Lakshmi T. *In vitro* anti arthritic activity of grape seed ethanolic extract. Int J Pharmacogn Phytochem Res. 2015;7(5):977–9.

[22] Djemaa-Landri K, Hamri-Zeghichi S, Valls J, Cluzet S, Tristan R, Boulahbal N, et al. Phenolic content and antioxidant activities of *Vitis vinifera* L. leaf extracts obtained by conventional solvent and microwave-assisted extractions. J Food Meas Charact. 2020;14:3551–64.

[23] Dong Q, Han J, Wu S, Pang Y, Chen Y, Ji Q, et al. Seasonal changes in the phenolic compound contents and bioactivities in *Betula platyphylla* bark determined by soil properties. Flora. 2024;317:152559.

[24] El Sawi SA, Elbatanony MM, El-Feky AM, Farghaly AA. Antimutagenic and Cytotoxic Potential of Punica granatum L. and Opuntia ficusindica L. Peels. Egyptian Journal of Chemistry. 2024;67(1):267–83.

[25] El-Ezz SF, Al-Harbi NA, Al-Qahtani SM, Allam HM, Abdein MA, Abdelgawad ZA. A comparison of the effects of several foliar forms of magnesium fertilization on “Superior Seedless” (*Vitis vinifera* L.) in saline soils. Coatings. 2022;12(2):201.

[26] El-Feky AM, El-Rashedy AA. Sterols and flavonoids in strawberry calyx with free radical scavenging, anti-inflammatory, and molecular dynamic study. Beni-Suef Univ J Basic Appl Sci. 2023;12:108.

[27] El-Feky AM, Aboulthana WM, El-Rashedy AA. Assessment of the *in vitro* anti-diabetic activity with molecular dynamic simulations of limonoids isolated from Adalia lemon peels. Sci Rep. 2024;14(1):21478. 10.1038/s41598-024-71198-5 PMC11401861 39277638

[28] El-Feky AM, Aboul Naser AF, Hamed MA. *Solanum melongena* Peels against Ethanol Induced Gastric Ulcer in Rats via Regulating Mucosal Enzymes, Oxidative Stress and Inflammatory Mediators’ Pathways. Egypt J Chem. 2024;67(10):587–600.

[29] El-Feky AM, Mohammed NA. Potential antioxidant and cytotoxic impacts of defatted extract rich in flavonoids from *Styphnolobium japonicum* leaves growing in Egypt. Sci Rep. 2024;14(1):18690. 10.1038/s41598-024-68675-2 PMC11319774 39134561

[30] Ellman GL, Courtney KD, Andres VJ, Featherstone RM. A new and rapid colorimetric determination of acetylcholinesterase activity. Biochem Pharmacol. 1961;7:88–95. 10.1016/0006-2952(61)90145-9 13726518

[31] ElNaker NA, Yousef AF, Yousef LF. A review of *Arthrocnemum* (*Arthrocaulon*) *macrostachyum* chemical content and bioactivity. Phytochem Rev. 2020;19:1427–48.

[32] El-Sayed MA, Al-Gendy AA, Hamdan DI, El-Shazly AM. Phytoconstituents, LC-ESI-MS profile, antioxidant and antimicrobial activities of Citrus x limon L. Burm. f. cultivar variegated pink lemon. Journal of Pharmaceutical Sciences and Research. 2017;9(4):375.

[33] El Sawi S, El-Feky AM, Younis EA, Farghaly AA, Hassan EE, El-Sayed AF. Flavonoid-enriched fractions of Pimpinella anisum and Coriandrum sativum: antioxidant, hepatoprotective, and antimutagenic activities supported by in vivo and dynamics simulation studies. Beni-Suef University Journal of Basic and Applied Sciences. 2026;15(1):47.

[34] El Sawi S, El-Feky AM, Elbatanony MM, El-khonezy MI, Aly HF. Phytochemical profiling of three Egyptian Phoenix dactylifera L cultivars’ leaves with comparative positive impacts in controlling the streptozotocin diabetic rats. Egyptian Pharmaceutical Journal. 2024;23(4):654–69.

[35] El-Shora H, Messgo SM, Ibrahim ME, Alfakharany MW. Purification and characterization of α-glucosidase from *Penicillium chrysogenum*. Int J Phytomedicine. 2018;10(4):175–80.

[36] Filimon RV, Rotaru L, Filimon RM. Quantitative investigation of leaf photosynthetic pigments during annual biological cycle of *Vitis vinifera* L. table grape cultivars. S Afr J Enol Vitic. 2016;37(1):1–4.

[37] Gashu K, Song C, Dubey AK, Acuña T, Sagi M, Agam N, et al. The Effect of Topo-Climate Variation on the Secondary Metabolism of Berries in White Grapevine Varieties (*Vitis vinifera*). Front Plant Sci. 2022;13:847268. 10.3389/fpls.2022.847268 PMC8958008 35350300

[38] Ghosh S, More P, Derle A, Patil AB, Markad P, Asok A, et al. Novel hit for the treatment of type II diabetes Mellitus with inhibitory activity against α-amylase and α-glucosidase. PlosOne. 2014;9(9):e106039. 10.1371/journal.pone.0106039 PMC4162539 25216353

[39] Glevitzky I, Dumitrel GA, Glevitzky M, Pasca B, Otrisal P, Bungau S, et al. Statistical analysis of the relationship between antioxidant activity and the structure of flavonoid compounds. Rev Chim. 2019;70:3103–7.

[40] Gouot JC, Smith JP, Holzapfel BP, Walker AR, Barril C. Grape berry flavonoids: a review of their biochemical responses to high and extreme high temperatures. J Exp Bot. 2019;70(2):397–423. 10.1093/jxb/ery392 30388247

[41] Grassi F, De Lorenzis G. Back to the origins: background and perspectives of grapevine domestication. Int J Mol Sci. 2021;22(9):4518. 10.3390/ijms22094518 PMC8123694 33926017

[42] Gülcü M, Ghafoor K, Al-Juhaimi F, Özcan MM, Uslu N, Babiker EE, et al. Effect of grape (*Vitis vinifera* L.) varieties and harvest periods on bioactive compounds, antioxidant activity, phenolic composition, mineral contents, and fatty acid compositions of *Vitis* leave and oils. J Food Process Preserv. 2020;44(11):e14890.

[43] Hashimoto H, Sugai Y, Uragami C, Gardiner AT, Cogdell RJ. Natural and artificial light-harvesting systems utilizing the functions of carotenoids. J Photochem Photobiol C Photochem Rev. 2015;25:46–70.

[44] Hassan AS, Awad HM, Magd-El-Din AA, Hafez TS. Synthesis and *in vitro* antitumor evaluation of novel Schiff bases. Med Chem Res. 2018;27:915–27.

[45] Hörtensteiner S, Kräutler B. Chlorophyll breakdown in higher plants. Biochim Biophys Acta. 2011;1807:977–88. 10.1016/j.bbabio.2010.12.007 21167811

[46] Huang Y, Zhang B, Li J, Liu H, Zhang Y, Yang Z, et al. Design, synthesis, biological evaluation and docking study of novel indole-2-amide as anti-inflammatory agents with dual inhibition of COX and 5-LOX. Eur J Med Chem. 2019;180:41–50. 10.1016/j.ejmech.2019.07.004 31299586

[47] Hussien AG, Fyiad AA, Ali MM, Mahmoud AE. The Influence of *Syzygium aromaticum* Ethanolic Extract against Streptozotocin-Induced Diabetic Rats. Egypt J Chem. 2024;67(2):357–69.

[48] Kabtni S, Sdouga D, Bettaib Rebey I, Save M, Trifi-Farah N, Fauconnier ML, et al. Influence of climate variation on phenolic composition and antioxidant capacity of *Medicago minima* populations. Sci Rep. 2020;10(1):8293. 10.1038/s41598-020-65160-4 PMC7237653 32427946

[49] Kalita D, Holm DG, LaBarbera DV, Petrash JM, Jayanty SS. Inhibition of α-glucosidase, α-amylase, and aldose reductase by potato polyphenolic compounds. PLoS ONE. 2018;13(1):e0191025. 10.1371/journal.pone.0191025 PMC5784920 29370193

[50] Kicel A, Owczarek A, Gralak P, Ciszewski P, Olszewska MA. Polyphenolic profile, antioxidant activity, and pro-inflammatory enzymes inhibition of leaves, flowers, bark and fruits of cotoneaster integerrimus: A comparative study. Phytochem Lett. 2019;30:349–55.

[51] Labanca F, Faraone I, Nolè MR, Hornedo-Ortega R, Russo D, García-Parrilla MC, et al. New insights into the exploitation of *Vitis vinifera* L. cv. Aglianico leaf extracts for nutraceutical purposes. Antioxidants (Basel). 2020;9(8):708. 10.3390/antiox9080708 PMC7463595 32759838

[52] Laemmli UK. Cleavage of structural proteins during the assembly of the head of bacteriophage T4. Nature. 1970;227(5259):680–5. 10.1038/227680a0 5432063

[53] Lu H, Xie T, Wu Q, Hu Z, Luo Y, Luo F. Alpha-glucosidase inhibitory peptides: sources, preparations, identifications, and action mechanisms. Nutrients. 2023;15(19):4267. 10.3390/nu15194267 PMC10574726 37836551

[54] Mabrouk AA, Tadros MI, El-Refaie WM. Improving the efficacy of Cyclooxegenase-2 inhibitors in the management of oral cancer: insights into the implementation of nanotechnology and mucoadhesion. J Drug Deliv Sci Technol. 2021;61:102240.

[55] Maleš Ž, Plazibat M, Vundać VB, Žuntar I, Pilepić KH. Thin-layer chromatographic analysis of flavonoids, phenolic acids, and amino acids in some Croatian *Hypericum* taxa. J Planar Chromatogr-Mod TLC. 2004;17:280–5.

[56] Meera S, Ramaiah N, Kalidindi N. Illustration of anti-rheumatic mechanism of rheumavedic capsule. Saudi Pharm J. 2011;19(4):279–84. 10.1016/j.jsps.2011.07.002 PMC3745176 23960770

[57] Mukhopadhyay N, Shukla A, Makhal PN, Kaki VR. Natural product-driven dual COX-LOX inhibitors: overview of recent studies on the development of novel anti-inflammatory agents. Heliyon. 2023;9:e14569. 10.1016/j.heliyon.2023.e14569 PMC10068128 37020932

[58] Naglah AM, Almehizia AA, Al-Omar MA, Al-Wasidi AS, Mohamed MH, Alsobeai SM, et al. Investigations of *in vitro* anti-acetylcholinesterase, anti-diabetic, anti-inflammatory, and anti-cancer efficacy of garden cress (*Lepidium sativum* Linn.) seed extracts, as well as *in vivo* biochemical and hematological assays. Pharmaceutics. 2025;17(4):446. 10.3390/pharmaceutics17040446 PMC12030678 40284441

[59] Naglah AM, Almehizia AA, Al-Wasidi AS, Alharbi AS, Alqarni MH, Hassan AS, et al. Exploring the potential biological activities of pyrazole-based Schiff bases as anti-diabetic, anti-Alzheimer’s, anti-inflammatory, and cytotoxic agents: in vitro studies with computational predictions. Pharmaceuticals (Basel). 2024;17(5):655. 10.3390/ph17050655 PMC11125359 38794225

[60] Nallathambi R, Poulev A, Zuk JB, Raskin I. Proanthocyanidin-rich grape seed extract reduces inflammation and oxidative stress and restores tight junction barrier function in Caco-2 colon cells. Nutrients. 2020;12(6):1623. 10.3390/nu12061623 PMC7352846 32492806

[61] Naser AF, Aziz WM, El-Feky AM, Elbatanony MM, Nasr NN, Ahmed YR, et al. Potential therapeutic effects of interleukin-1 receptor type 1 antagonist and the ethyl acetate fraction of *Murraya exotica* leaves against κ-carrageenan induced vein thrombosis in rats. Phytomedicine Plus. 2025;5(2):100795.

[62] Nei M, Li WS. Mathematical model for studing genetic variation in terms of restriction endonuclease. Proc Natl Acad Sci U S A. 1979;76:5269–73. 10.1073/pnas.76.10.5269 PMC413122 291943

[63] Nowakowski AB, Wobig WJ, Petering DH. Native SDS-PAGE: high resolution electrophoretic separation of proteins with retention of native properties including bound metal ions. Metallomics. 2014;6(5):1068–78. 10.1039/c4mt00033a PMC4517606 24686569

[64] Ouattar H, Zouirech O, Kara M, Assouguem A, Almutairi SM, Al-Hemaid FM, et al. *In vitro* study of the phytochemical composition and antioxidant, immunostimulant, and hemolytic activities of *Nigella sativa* (Ranunculaceae) and *Lepidium sativum* seeds. Molecules. 2022;27(18):5946. 10.3390/molecules27185946 PMC9505328 36144678

[65] Oyaizu M. Studies on product of browning reaction prepared from glucose amine. Jpn J Nutr. 1986;44(6):307–15.

[66] Oyedapo OO, Famurewa AJ. Antiprotease and membrane stabilizing activities of extracts of *Fagara zanthoxyloides*, *Olax subscorpioides* and *Tetrapleura tetraptera*. Int J Pharmacogn. 1995;33(1):65–9.

[67] Pandey P, Khan F, Alzahrani FA, Qari HA, Oves M. A novel approach to unraveling the apoptotic potential of rutin (bioflavonoid) via targeting Jab1 in cervical cancer cells. Molecules. 2021;26(18):5529. 10.3390/molecules26185529 PMC8472561 34577000

[68] Perera HDSM, Samarasekera JKRR, Handunnetti SM, Weerasena OVDSJ, Weeratunga HD, Jabeen A, et al. In vitro pro-inflammatory enzyme inhibition and anti-oxidant potential of selected sri lankan medicinal plants. BMC Complementary Altern Med. 2018;18:271. 10.1186/s12906-018-2335-1 PMC6169004 30285710

[69] Popescu M, Radivojevic K, Trasca D-M, Varut RM, Enache I, Osman A. Natural antidiabetic agents: insights into Ericaceae-derived phenolics and their role in metabolic and oxidative modulation in diabetes. Pharmaceuticals (Basel). 2025;18(5):682. 10.3390/ph18050682 PMC12115297 40430501

[70] Prieto P, Pineda M, Aguilar M. Spectrophotometric quantitation of antioxidant capacity through the formation of a phosphomolybdenum complex: specific application to the determination of vitamin E. Anal Biochem. 1999;269:337–41. 10.1006/abio.1999.4019 10222007

[71] Quan TH, Benjakul S, Sae-leaw T, Balange AK, Maqsood S. Protein–polyphenol conjugates: antioxidant property, functionalities and their applications. Trends Food Sci Technol. 2019;91:507–17.

[72] Rahman MM, Islam MB, Biswas M, Alam AK. *In vitro* antioxidant and free radical scavenging activity of different parts of *Tabebuia pallida* growing in Bangladesh. BMC Res Notes. 2015;8(1):621–8. 10.1186/s13104-015-1618-6 PMC4627625 26518275

[73] Rammesmayer G, Praznik W. Fast and sensitive simultaneous staining method of Q-enzyme, α-amylase, R-enzyme, phosphorylase and soluble starch synthase separated by starch: polyacrylamide gel electrophoresis. J Chromatogr. 1992;623(2):399–402.

[74] Rana A, Kaur J, Sharma K, Singh J, Bhadariya V. A comprehensive review on the nutritional value and health benefits of grape leaves. Pharm Innov J. 2022;1(6):2235–43.

[75] Rotaru L, Filimon VR, Filimon RM, Mustea M, Bernardis RR, Colibaba LC. Preliminary studies on some morpho-structural and biochemical characterization of some genotypes of *Vitis vinifera* L. cultivated in northeast Romania. Journal of Applied Life Sciences and Environment. 2024;57(1):69–90.

[76] Sargolzaei M, Rustioni L, Cola G, Ricciardi V, Bianco PA, Maghradze D, et al. Georgian Grapevine Cultivars: Ancient Biodiversity for Future Viticulture. Front Plant Sci. 2021;12:630122. 10.3389/fpls.2021.630122 PMC7892605 33613611

[77] Schumacher I, Menghini D, Ovinnikov S, Hauenstein M, Fankhauser N, Zipfel C, et al. Evolution of chlorophyll degradation is associated with plant transition to land. Plant J. 2022;109(6):1473–88. 10.1111/tpj.15645 PMC9306834 34931727

[78] Shaikh IA, Alshabi AM, Alkahtani SA, Orabi MAA, Abdel-Wahab BA, Walbi IA, et al. Apoptotic cell death via activation of DNA degradation, caspase-3 activity, and suppression of Bcl-2 activity: an evidence-based *Citrullus colocynthis* cytotoxicity mechanism toward MCF-7 and A549 cancer cell lines. Separations. 2022;9:411.

[79] Singh J, Rasane P, Kaur R, Kaur H, Garg R, Kaur S, et al. Valorization of grape (*Vitis vinifera*) leaves for bioactive compounds: novel green extraction technologies and food-pharma applications. Front Chem. 2023;11:1290619. 10.3389/fchem.2023.1290619 PMC10754528 38156021

[80] Singleton VL, Rossi JA. Colorimetry of total phenolics with phosphomolybdicphosphotungstic acid reagents. Am J Enol Vitic. 1965;16(3):144–58.

[81] Sinha SK, Prasad SK, Islam MA, Gurav SS, Patil RB, AlFaris NA, et al. Identification of bioactive compounds from *Glycyrrhiza glabra* as possible inhibitor of SARS-CoV-2 spike glycoprotein and non-structural protein-15: a pharmacoinformatics study. J Biomol Struct Dyn. 2021;39(13):4686–700. 10.1080/07391102.2020.1779132 PMC7309308 32552462

[82] Solaiman MM. Agglutination effect of selected medicinal plant leaf crude extracts on ABO blood group. Am J Plant Biol. 2021;6:11–8.

[83] Taskesenlioglu MY, Ercisli S, Kupe M, Ercisli N. History of grape in Anatolia and historical sustainable grape production in Erzincan agroecological conditions in Turkey. Sustainability. 2022;14(3):1496.

[84] Vichai V, Kirtikara K. Sulforhodamine B colorimetric assay for cytotoxicity screening. Nat Protoc. 2006;1(3):1112–6. 10.1038/nprot.2006.179 17406391

[85] Yadav P, Yadav R, Jain S, Vaidya A. Caspase-3: a primary target for natural and synthetic compounds for cancer therapy. Chem Biol Drug Des. 2021;98:144–65. 10.1111/cbdd.13860 33963665

[86] Yang B, He S, Liu Y, Liu B, Ju Y, Kang D, et al. Transcriptomics integrated with metabolomics reveals the effect of regulated deficit irrigation on anthocyanin biosynthesis in Cabernet Sauvignon grape berries. Food Chem. 2020;314:126170. 10.1016/j.foodchem.2020.126170 31978717

[87] Zhang H, Zhang F, Wu F, Guo L, Xu X. Purification and characterization of endogenous α-amylase from glutinous rice flour. Foods. 2025;14(10):1679. 10.3390/foods14101679 PMC12110810 40428461

[88] Zhang JL, Li XG, Xu XH, Chen HP, Li YL, Guy RD. Leaf morphology, photosynthesis and pigments change with age and light regime in savin juniper. 2021;23(6): 1097–1108. 10.1111/plb.13256 33756015

[89] Zhang JY, Pan DL, Jia ZH, Wang T, Wang G, Guo ZR. Chlorophyll, carotenoid and vitamin C metabolism regulation in *Actinidia chinensis* “Hongyang” outer pericarp during fruit development. PLoS ONE. 2018;13(3):e0194835. 10.1371/journal.pone.0194835 PMC5868826 29579114

