# Supplementary material for: Harvest-Time–Dependent variations in phytochemistry and in vitro bioactivities of Vitis vinifera Leaves
Source: BMC Complement Med Ther. 2026 Sep 26;26:278. doi: 10.1186/s12906-026-05578-x (PMC13615614; doi:10.1186/s12906-026-05578-x)
Supplement: Supplementary file 1 [file 12906_2026_5578_MOESM1_ESM.docx]

**Supplementary file**

**Supplementary Fig. 1.** Mass spectrum of *p*-coumaric acid.

**Supplementary Fig. 2.** ^1^H-NMR spectrum of *p*-coumaric acid.

**Supplementary Fig. 3.** ^13^C-NMR spectrum of *p*-coumaric acid.

**Supplementary Fig. 4.** Mass spectrum of isorhamnetin.

**Supplementary Fig. 5.** ^1^H-NMR spectrum of isorhamnetin.

**Supplementary Fig. 6.** ^13^C-NMR spectrum of isorhamnetin.

**Supplementary Fig. 7.** Unprocessed α-amylase isoenzyme pattern.

**Supplementary Fig. 8.** Unprocessed α-amylase isoenzyme pattern (Analyzed Image).

**Supplementary Fig. 9.** Unprocessed α-glucosidase isoenzyme pattern.

**Supplementary Fig. 10.** Unprocessed α-glucosidase isoenzyme pattern (Analyzed Image).

**Supplementary Table 1:** Native electrophoretic α-amylase isoenzymes pattern showing the anti-diabetic activity of extract from *V. vinifera* leaves harvested at three different times compared to Acarbose (standard) on the physiological state of α-amylase enzyme.

|  | **Enzyme** | | | **Enzyme treated with** | | | | | | | | | | | |
| --- | --- | --- | --- | --- | --- | --- | --- | --- | --- | --- | --- | --- | --- | --- | --- |
|  |  |  |  | **STD** | | | **March** | | | **May** | | | **September** | | |
|  | **Rf** | **Qty** | **B.%** | **Rf** | **Qty** | **B.%** | **Rf** | **Qty** | **B.%** | **Rf** | **Qty** | **B.%** | **Rf** | **Qty** | **B.%** |
| **α-amy1** | **0.28** | **22.12** | **53.46** | **-** | **-** | **-** | **0.28** | **20.82** | **100.00** | **-** | **-** | **-** | **-** | **-** | **-** |
| **-** | **-** | **-** | **-** | **0.36** | **17.32** | **100.00** | **-** | **-** | **-** | **-** | **-** | **-** | **-** | **-** | **-** |
| **α-amy2** | **0.55** | **19.26** | **46.54** | **-** | **-** | **-** | **-** | **-** | **-** | **-** | **-** | **-** | **0.56** | **18.66** | **100.00** |
| **-** | **-** | **-** | **-** | **-** | **-** | **-** | **-** | **-** | **-** | **0.64** | **21.88** | **100.00** | **-** | **-** | **-** |
| **SI%** | **-** | | | **0.00** | | | **66.67** | | | **0.00** | | | **66.67** | | |
| **Diff%** | **-** | | | **100.00** | | | **33.33** | | | **100.00** | | | **33.33** | | |

**Rf.:** Relative Mobility, **Qty:** Band Quantity, **B%:** Band Percent, **SI%:** Similarity Percent, **Diff%:** Difference Percent.

**Supplementary Table 2:** Native electrophoretic protein pattern showing the anti-diabetic activity of extract from *V. vinifera* leaves harvested at three different times on the quantity of the α-glucosidase enzyme checked by SDS PAGE compared to Acarbose (standard).

| **Mwts**  **(KDa)** | **Enzyme** | | | **Enzyme treated with** | | | | | | | | | | | | **Marker** | | |
| --- | --- | --- | --- | --- | --- | --- | --- | --- | --- | --- | --- | --- | --- | --- | --- | --- | --- | --- |
|  |  |  |  | **STD** | | | **March** | | | **May** | | | **September** | | |  |  |  |
|  | **Rf.** | **Mwt** | **Qty** | **Rf.** | **Mwt** | **Qty** | **Rf.** | **Mwt** | **Qty** | **Rf.** | **Mwt** | **Qty** | **Rf.** | **Mwt** | **Qty** | **Rf.** | **Mwt** | **Qty** |
| **66.00** | **-** | **-** | **-** | **-** | **-** | **-** | **-** | **-** | **-** | **-** | **-** | **-** | **-** | **-** | **-** | **0.21** | **66.00** | **8.31** |
|  | **0.40** | **51.08** | **20.47** | **-** | **-** | **-** | **0.41** | **50.94** | **17.27** | **-** | **-** | **-** | **0.40** | **51.36** | **14.26** | **-** | **-** | **-** |
| **45.00** | **-** | **-** | **-** | **-** | **-** | **-** | **-** | **-** | **-** | **-** | **-** | **-** | **-** | **-** | **-** | **0.49** | **45.00** | **9.07** |
| **-** | **-** | **-** | **-** | **-** | **-** | **-** | **-** | **-** | **-** | **0.53** | **43.99** | **10.20** | **-** | **-** | **-** | **-** | **-** | **-** |
| **-** | **-** | **-** | **-** | **0.61** | **41.36** | **5.67** | **-** | **-** | **-** | **-** | **-** | **-** | **-** | **-** | **-** | **-** | **-** | **-** |
| **36.00** | **-** | **-** | **-** | **-** | **-** | **-** | **-** | **-** | **-** | **-** | **-** | **-** | **-** | **-** | **-** | **0.81** | **36.00** | **7.80** |
| **Diff%** | **-** | | | **72.29** | | | **15.64** | | | **50.19** | | | **30.35** | | | **-** | | |

**Rf.:** Relative Mobility, **Mwt:** Molecular Weight, **Qty:** Band Quantity, **Diff%:** Difference Percent.

**Supplementary Table 3:** The *in vitro* cytotoxic activity of extract from *V. vinifera* leaves harvested at three different harvest times against human hepatocellular carcinoma cell lines (HEPG-2) and compared to Doxorubicin used as standard.

| **March** | | | | | | |
| --- | --- | --- | --- | --- | --- | --- |
| **Conc.** | **0.00** | **31.13** | **62.50** | **125.00** | **250.00** | **500.00** |
| OD | 0.26 | 0.22 | 0.19 | 0.13 | 0.08 | 0.06 |
|  | 0.27 | 0.21 | 0.20 | 0.14 | 0.08 | 0.04 |
|  | 0.25 | 0.23 | 0.21 | 0.13 | 0.08 | 0.04 |
| Mean | 0.26 | 0.22 | 0.20 | 0.13 | 0.08 | 0.05 |
| Viability % | 100.00 | 85.19 | 71.92 | 52.29 | 47.67 | 20.88 |
| Cytotoxicity % | 0.00 | 14.81 | 28.08 | 47.71 | 52.33 | 79.12 |
| **IC_50_** | **167.00 ± 3.79 μg/mL** | | | | | |

| **May** | | | | | | |
| --- | --- | --- | --- | --- | --- | --- |
| **Conc.** | **0.00** | **6.25** | **12.50** | **25.00** | **50.00** | **100.00** |
| OD | 0.32 | 0.27 | 0.24 | 0.17 | 0.12 | 0.01 |
|  | 0.34 | 0.27 | 0.24 | 0.15 | 0.12 | 0.02 |
|  | 0.32 | 0.28 | 0.23 | 0.17 | 0.11 | 0.02 |
| Mean | 0.33 | 0.28 | 0.24 | 0.16 | 0.12 | 0.02 |
| Viability % | 100.00 | 64.83 | 54.71 | 39.20 | 29.86 | 14.78 |
| Cytotoxicity % | 0.00 | 35.17 | 45.29 | 60.80 | 70.14 | 85.22 |
| **IC_50_** | **24.53 ± 2.62 μg/mL** | | | | | |

| **September** | | | | | | |
| --- | --- | --- | --- | --- | --- | --- |
| **Conc.** | **0.00** | **6.25** | **12.50** | **25.00** | **50.00** | **100.00** |
| OD | 0.25 | 0.21 | 0.18 | 0.13 | 0.07 | 0.06 |
|  | 0.26 | 0.20 | 0.19 | 0.14 | 0.07 | 0.04 |
|  | 0.24 | 0.22 | 0.20 | 0.13 | 0.08 | 0.04 |
| Mean | 0.25 | 0.21 | 0.19 | 0.13 | 0.08 | 0.05 |
| Viability % | 100.00 | 66.53 | 58.58 | 46.12 | 34.97 | 14.65 |
| Cytotoxicity % | 0.00 | 33.47 | 41.42 | 53.88 | 65.03 | 85.35 |
| **IC_50_** | **45.21 ± 2.58 μg/mL** | | | | | |

| **Doxorubicin** | | | | | | |
| --- | --- | --- | --- | --- | --- | --- |
| **Conc.** | **0.00** | **6.25** | **12.50** | **25.00** | **50.00** | **100.00** |
| OD | 0.20 | 0.16 | 0.13 | 0.10 | 0.07 | 0.59 |
|  | 0.20 | 0.17 | 0.12 | 0.10 | 0.06 | 0.57 |
|  | 0.19 | 0.17 | 0.13 | 0.09 | 0.07 | 0.57 |
| Mean | 0.20 | 0.16 | 0.12 | 0.09 | 0.07 | 0.58 |
| Viability % | 100.00 | 62.51 | 53.58 | 28.53 | 14.63 | 8.66 |
| Cytotoxicity % | 0.00 | 37.49 | 46.42 | 71.47 | 85.37 | 91.34 |
| **IC_50_** | **14.38 ± 0.86 μg/mL** | | | | | |

**Supplementary Table 4:** The *in vitro* cytotoxic activity of extract from *V. vinifera* leaves harvested at three different harvest times against human colon cancer cell lines (CACO-2) and compared to Doxorubicin used as standard.

| **March** | | | | | | |
| --- | --- | --- | --- | --- | --- | --- |
| **Conc.** | **0.00** | **31.13** | **62.50** | **125.00** | **250.00** | **500.00** |
| OD | 0.27 | 0.23 | 0.20 | 0.14 | 0.08 | 0.07 |
|  | 0.28 | 0.22 | 0.20 | 0.15 | 0.08 | 0.05 |
|  | 0.26 | 0.24 | 0.22 | 0.14 | 0.09 | 0.05 |
| Mean | 0.27 | 0.23 | 0.21 | 0.14 | 0.09 | 0.06 |
| Viability % | 100.00 | 70.02 | 59.18 | 48.85 | 37.28 | 16.20 |
| Cytotoxicity % | 0.00 | 29.98 | 40.82 | 51.15 | 62.72 | 83.80 |
| **IC_50_** | **153.26 ± 3.57 μg/mL** | | | | | |

| **May** | | | | | | |
| --- | --- | --- | --- | --- | --- | --- |
| **Conc.** | **0.00** | **6.25** | **12.50** | **25.00** | **50.00** | **100.00** |
| OD | 0.26 | 0.22 | 0.18 | 0.11 | 0.07 | 0.04 |
|  | 0.28 | 0.22 | 0.18 | 0.10 | 0.07 | 0.05 |
|  | 0.26 | 0.22 | 0.18 | 0.11 | 0.06 | 0.05 |
| Mean | 0.27 | 0.22 | 0.18 | 0.11 | 0.07 | 0.05 |
| Viability % | 100.00 | 70.21 | 51.55 | 37.40 | 28.56 | 14.27 |
| Cytotoxicity % | 0.00 | 29.79 | 48.45 | 62.60 | 71.44 | 85.73 |
| **IC_50_** | **37.73 ± 3.57 μg/mL** | | | | | |

| **September** | | | | | | |
| --- | --- | --- | --- | --- | --- | --- |
| **Conc.** | **0.00** | **6.25** | **12.50** | **25.00** | **50.00** | **100.00** |
| OD | 0.26 | 0.22 | 0.19 | 0.14 | 0.08 | 0.07 |
|  | 0.27 | 0.21 | 0.20 | 0.14 | 0.08 | 0.05 |
|  | 0.25 | 0.23 | 0.21 | 0.14 | 0.09 | 0.05 |
| Mean | 0.26 | 0.22 | 0.20 | 0.14 | 0.08 | 0.06 |
| Viability % | 100.00 | 67.49 | 53.12 | 47.09 | 35.93 | 15.62 |
| Cytotoxicity % | 0.00 | 32.51 | 46.88 | 52.91 | 64.07 | 84.38 |
| **IC_50_** | **57.90 ± 3.60 μg/mL** | | | | | |

| **Doxorubicin** | | | | | | |
| --- | --- | --- | --- | --- | --- | --- |
| **Conc.** | **0.00** | **6.25** | **12.50** | **25.00** | **50.00** | **100.00** |
| OD | 0.28 | 0.24 | 0.18 | 0.11 | 0.13 | 0.03 |
|  | 0.29 | 0.24 | 0.17 | 0.10 | 0.12 | 0.01 |
|  | 0.27 | 0.24 | 0.16 | 0.10 | 0.13 | 0.03 |
| Mean | 0.28 | 0.24 | 0.17 | 0.11 | 0.12 | 0.02 |
| Viability % | 100.00 | 59.51 | 50.30 | 41.52 | 31.68 | 13.77 |
| Cytotoxicity % | 0.00 | 40.49 | 49.70 | 58.48 | 68.32 | 86.23 |
| **IC_50_** | **30.70 ± 2.65 μg/mL** | | | | | |

**Supplementary Table 5:** The *in vitro* cytotoxic activity of extract from *V. vinifera* leaves harvested at three different harvest times against human lung colon cancer cell lines (A549) and compared to Doxorubicin used as standard.

| **March** | | | | | | |
| --- | --- | --- | --- | --- | --- | --- |
| **Conc.** | **0.00** | **31.13** | **62.50** | **125.00** | **250.00** | **500.00** |
| OD | 0.27 | 0.22 | 0.19 | 0.13 | 0.08 | 0.06 |
|  | 0.27 | 0.22 | 0.20 | 0.14 | 0.08 | 0.04 |
|  | 0.26 | 0.23 | 0.22 | 0.13 | 0.09 | 0.04 |
| Mean | 0.27 | 0.22 | 0.20 | 0.14 | 0.08 | 0.05 |
| Viability % | 100.00 | 71.01 | 47.80 | 22.97 | 17.54 | 8.77 |
| Cytotoxicity % | 0.00 | 28.99 | 52.20 | 77.03 | 82.46 | 91.23 |
| **IC_50_** | **368.97 ± 13.10 μg/mL** | | | | | |

| **May** | | | | | | |
| --- | --- | --- | --- | --- | --- | --- |
| **Conc.** | **0.00** | **31.13** | **62.50** | **125.00** | **250.00** | **500.00** |
| OD | 0.35 | 0.31 | 0.27 | 0.20 | 0.16 | 0.06 |
|  | 0.37 | 0.31 | 0.27 | 0.19 | 0.16 | 0.07 |
|  | 0.35 | 0.31 | 0.27 | 0.20 | 0.15 | 0.07 |
| Mean | 0.36 | 0.31 | 0.27 | 0.20 | 0.16 | 0.06 |
| Viability % | 100.00 | 50.73 | 37.25 | 27.02 | 20.64 | 10.31 |
| Cytotoxicity % | 0.00 | 49.27 | 62.75 | 72.98 | 79.36 | 89.69 |
| **IC_50_** | **105.82 ± 3.53 μg/mL** | | | | | |

| **September** | | | | | | |
| --- | --- | --- | --- | --- | --- | --- |
| **Conc.** | **0.00** | **31.13** | **62.50** | **125.00** | **250.00** | **500.00** |
| OD | 0.25 | 0.21 | 0.18 | 0.13 | 0.07 | 0.06 |
|  | 0.26 | 0.20 | 0.19 | 0.14 | 0.07 | 0.04 |
|  | 0.24 | 0.22 | 0.20 | 0.13 | 0.08 | 0.04 |
| Mean | 0.25 | 0.21 | 0.19 | 0.13 | 0.08 | 0.05 |
| Viability % | 100.00 | 59.68 | 43.82 | 31.79 | 24.28 | 12.13 |
| Cytotoxicity % | 0.00 | 40.32 | 56.18 | 68.21 | 75.72 | 87.87 |
| **IC_50_** | **216.31 ± 2.55 μg/mL** | | | | | |

| **Doxorubicin** | | | | | | |
| --- | --- | --- | --- | --- | --- | --- |
| **Conc.** | **0.00** | **6.25** | **12.50** | **25.00** | **50.00** | **100.00** |
| OD | 0.20 | 0.16 | 0.13 | 0.10 | 0.07 | 0.05 |
|  | 0.20 | 0.17 | 0.12 | 0.10 | 0.06 | 0.04 |
|  | 0.19 | 0.17 | 0.13 | 0.09 | 0.07 | 0.04 |
| Mean | 0.20 | 0.16 | 0.12 | 0.09 | 0.07 | 0.04 |
| Viability % | 100.00 | 51.86 | 40.63 | 19.52 | 14.91 | 7.45 |
| Cytotoxicity % | 0.00 | 48.14 | 59.37 | 80.48 | 85.09 | 92.55 |
| **IC_50_** | **54.79 ± 2.86 μg/mL** | | | | | |

**Supplementary Table 6:** The *in vitro* cytotoxic activities of extract from *V. vinifera* leaves harvested at three different times against human hepatocellular carcinoma (HEPG-2), colon (CACO-2) and lung (A549) cancer cell lines.

| **Harvest time** | **Median Inhibitory Concentration (**IC_50_**)**  **(**µg/mL**)** | | |
| --- | --- | --- | --- |
|  | **HEPG-2** | **CACO-2** | **A549** |
| **March** | 167.00 ± 3.79 | 153.26 ± 3.57 | 368.97 ± 13.10 |
| **May** | **24.53 ± 2.62*** | **37.73 ± 3.57*** | **105.82 ± 3.53*** |
| **September** | 45.21 ± 2.58 | 57.90 ± 3.60 | 216.31 ± 2.55 |
| **DOX** | **14.38 ± 0.86** | **30.70 ± 2.65** | **54.79 ± 2.86** |

*Denotes the extract with the highest cytotoxic activity. The data was calculated from n=3/extract and shown as mean ± SE.

**Supplementary Table 7:** The enzymatic assay values after the treatment of human hepatocellular carcinoma (HepG-2), colon (CACO-2) and lung (A549) cancer cells with of extract from *V. vinifera* leaves harvested at three different times.

| **Harvest time** | **Median Inhibitory Concentration (**IC_50_**) (**µg/mL**)** | | | | | |
| --- | --- | --- | --- | --- | --- | --- |
|  | **HepG-2** | | **CACO-2** | | **A549** | |
|  | **Caspase-3 (**pg/mL**)** | **Bcl-2**  **(**ng/mL**)** | **Caspase-3 (**pg/mL**)** | **Bcl-2**  **(**ng/mL**)** | **Caspase-3 (**pg/mL**)** | **Bcl-2**  **(**ng/mL**)** |
| **DMSO** | 114.13 ± 0.76 | 7.60 ± 0.03 | 85.60 ± 0.57 | 10.13 ± 0.04 | 98.44 ± 0.66 | 8.81 ± 0.03 |
| **March** | 119.84 ± 0.80 | 7.24 ± 0.03 | 89.88 ± 0.60 | 9.65 ± 0.03 | 103.36 ± 0.69 | 8.39 ± 0.03 |
| **May** | **211.14 ± 1.41*** | **4.11 ± 0.01*** | **158.36 ± 1.06*** | **5.48 ± 0.02*** | **182.11 ± 1.22*** | **4.76 ± 0.02*** |
| **September** | 171.20 ± 1.14 | 6.08 ± 0.02 | 128.40 ± 0.86 | 8.11 ± 0.03 | 147.66 ± 0.99 | 7.05 ± 0.03 |
| **DOX** | **257.65 ± 1.72** | **3.37 ± 0.01** | **193.24 ± 1.29** | **4.49 ± 0.02** | **222.22 ± 1.49** | **3.90 ± 0.01** |

*Denotes the extract with the highest cytotoxic activity. The data was calculated from n=3/extract and shown as mean ± SE.
